# Host–microbiome interplay supports heat stress resilience in zebu calves

**DOI:** 10.1186/s42523-026-00539-8

**Published:** 2026-03-03

**Authors:** Brijesh Yadav, Goutam Banerjee, Anandita Srivastava, Arun Kumar Madan, Ravindra Kumar, Pratik Banerjee

**Affiliations:** 1 Prof. M.D. Pandey Bio-Climatology Laboratory, Department of Veterinary Physiology, College of Veterinary Science and Animal Husbandry, Veterinary University (DUVASU), Mathura, UP India; 2 Food Safety and Molecular Microbiology Laboratory, Department of Food Science and Human Nutrition, University of Illinois Urbana-Champaign, Urbana, IL USA; 3 ICAR-Central Institute for Research on Goats, Farah, Mathura, UP India

**Keywords:** Zebu calves, Heat stress, Rumen microbiome, Host–microbe interactions, Thermal resilience

## Abstract

This study delineates the impact of heat stress (HS) on rumen microbial diversity and its association with host physiological responses in zebu calves, aiming to elucidate the microbiome-mediated mechanisms underlying thermal resilience. Six zebu calves (8–11 months; 120–150 kg) were sequentially subjected to thermoneutral (TN) [temperature-humidity index (THI) of 72.32] and HS (THI of 94.82) conditions for 21 days each, with a 10-day acclimatization and a 45-day washout interval. Physiological assessment under HS revealed significant (*p <* 0.05) elevations in rectal temperature, respiratory rate, cortisol, and IL-6 levels, along with significant (*p <* 0.05) declines in plasma aspartate aminotransferase (AST) activity and triglyceride levels. The volatile fatty acid (VFA) concentration remained unaltered, suggesting functional stability of the fermentative activity during HS. HS significantly altered the microbial diversity in the rumen of zebu calves, especially among bacteria, fungi, and protozoa, with archaeal changes limited to alpha diversity. HS led to a shift toward Pseudomonadota (notably *Pseudomonas* and *Stenotrophomonas*), whereas the TN condition favored Bacillota (e.g., *Aerococcus* and *Soilbacillus*). The genus-level fungal profile exhibited a high abundance of *Thermothielavioides* in the HS group and *Schizosaccharomyces* in the TN group. Species-level profiling indicated the enrichment of *S. pombe* in both groups. Similarly, the species-level protozoal profiles indicated a high abundance of *Entodinium caudatum* in both groups. Although the archaeal composition remained largely stable, the relative abundance of *Methanobrevibacter* (particularly *M. ruminantium*) was more prevalent in both groups. This study also indicated that HS enriched folate and amino acid biosynthesis pathways, while TN favored glycolytic metabolism. Microbial taxa like *Stenotrophomonas* and *Aerococcus* correlated strongly with stress and metabolic markers like IL-6, cortisol, HSP-90, AST, and triglycerides. Despite the increased abundance of opportunistic taxa under HS, the maintenance of VFA homeostasis and strong taxa–phenotype associations underscore the integral role of the rumen microbiome in mediating host adaptation to HS, thereby contributing to the thermal resilience of zebu calves.

**Supplementary Information:**

The online version contains supplementary material available at 10.1186/s42523-026-00539-8.

## Introduction

Global climate change presents an escalating challenge to sustainable livestock production, with rising ambient temperatures and humidity leading to increased frequency and duration of HS and heat wave episodes, particularly in the tropical and subtropical regions. This climatic shift significantly impacts animal production, disease prevalence, and the availability of feed, fodder, and water, ultimately threatening global food and nutritional security (IPCC, 2023). Ruminants, especially dairy cattle, are highly susceptible to HS, which triggers a cascade of systemic changes compromising milk and meat productivity, product quality, and animal health and welfare [1–3]. Consequently, the capacity of livestock farming to support livelihoods and meet the burgeoning demand for animal products is increasingly imperiled.

In the face of these challenges, several short-, medium-, and long-term strategies are being explored for sustainable livestock production. In the Indian context, selective breeding and propagation of indigenous cattle are particularly promising approaches. Indian zebu cattle (*Bos indicus*) are renowned for their inherent climate resilience, exhibiting superior physiological threshold limits to HS owing to their distinctive anatomical, physiological, and behavioral adaptations [4–6]. Recent advancements in molecular biology have begun to decipher the complex genetic and epigenetic factors underpinning the remarkable HS tolerance of zebu cattle [7, 8]. Beyond their thermotolerance, zebu cattle are also recognized for their enhanced disease resistance, displaying reduced susceptibility to parasitic infestations and metabolic diseases compared to *Bos taurus* and crossbred cattle [6, 9]. Documented evidence consistently highlights the superior HS resistance of zebu cattle through differential physiological, biochemical, molecular, and production responses to thermal stress when compared to crossbred and temperate cattle [7].

The rumen, the most crucial compartment of the ruminant gastrointestinal tract, hosts a complex and dynamic ecosystem of bacteria, protozoa, and fungi. These microorganisms are indispensable for the fermentation of plant materials indigestible by monogastrics, synthesizing vital nutrients, and profoundly influencing animal productivity, immunity, health, and welfare. The rumen ecosystem maintains a delicate equilibrium between the rumen environment, its diverse microbiome, and host physiology to optimize ruminal fermentation [10]. HS perturbs host physiology, which in turn profoundly impacts the rumen ecosystem, potentially leading to a reprogramming of ruminal fermentation and subsequent alterations in production and health outcomes [11, 12]. Therefore, understanding the intricate responses of the rumen ecosystem, in addition to host physiological responses, is critical for unraveling the role of rumen microbes in acquiring thermotolerance in ruminants [13, 14].

Physiologically, HS leads to increased respiration rate, pulse rate, and rectal temperature, alongside alterations in hemogram, leucogram, liver function, metabolism, redox status, and endocrine function, ultimately compromising the immune system in ruminant livestock [3]. A decline in feed intake during HS is widely reported as a primary cause for changes in digestibility, volatile fatty acid (VFA) production, and energy utilization [5, 8, 15–18]. However, there is considerable variation in HS-mediated changes in rumen physiology among different ruminant species and even among breeds of the same species. While it is well-established that host responses to HS vary among ruminant species and breeds due to their differential genetic, morphological, anatomical, and physiological makeup, the precise reasons for differential responses in rumen physiology to HS remain less clear. Many researchers propose that HS-induced changes in rumen physiology are a complex outcome of the intricate interaction between host responses and the rumen ecosystem.

Recent metagenomic sequencing studies have consistently revealed that HS alters the microbial population in cattle [11, 19–26], but the resulting changes in VFA production and digestibility exhibit high variability among cattle breeds [20, 23, 27, 28]. Only a limited number of studies have explicitly reported that variations in functional responses of rumen physiology to HS may be attributed to differential microbial resilience among breeds and species. For example, Zhang et al. (2022) highlighted the importance of differences in gut microbes of cattle from mesotemperate to tropical climates for the acquisition of heat tolerance [25]. Similarly, goats [18, 29] and sheep [12] have evolved a suitable rumen microbiota to maximize digestion and absorption, thereby adapting to harsh environmental conditions. Wang et al. (2022) further suggested that specific changes in the rumen microbiota and metabolomics in heat-stressed cows could be associated with improved adaptability to HS [24]. Our previous work has also demonstrated that the resilience of rumen microbiota in buffalo exhibited adaptive responses by suitably altering their abundance to mitigate the adverse effects on fermentation [30].

While zebu cattle are demonstrably adapted to HS due to their robust physiological mechanisms, the specific contribution and underlying mechanisms of their ruminal microbiome in acquiring and maintaining this resilience to HS remain largely unexplored. There is a critical knowledge gap in understanding how the rumen microbiome of zebu cattle responds to HS and how these microbial alterations contribute to the host’s overall thermotolerance, particularly in calves, which are a vulnerable population. We hypothesize that the rumen microbiome of zebu cattle calves plays a significant role in their resilience to HS by undergoing adaptive shifts in its composition and metabolic functions, thereby contributing to the host’s physiological adaptability. The present study aims to comprehensively investigate the effect of HS on the rumen microbiome, metabolome, and physiological responses in zebu cattle calves to elucidate the functional host-microbiome interplay that supports HS resilience.

## Materials and methods

### Study location

The study was conducted at Prof. M.D. Pandey Bio-Climatology Laboratory of the Department of Veterinary Physiology, College of Veterinary Science and Animal Husbandry, DUVASU, Mathura, Uttar Pradesh. It is situated at a 27°N latitude and 78°E longitude and 176 m above mean sea level. The annual temperature ranges from 4 to 46 °C, while the relative humidity ranges from 25 to 85%. The annual rainfall in this area ranges from 200 to 400 mm with an inconsistent distribution throughout the year.

### Experimental animals

**Fig. 1:**
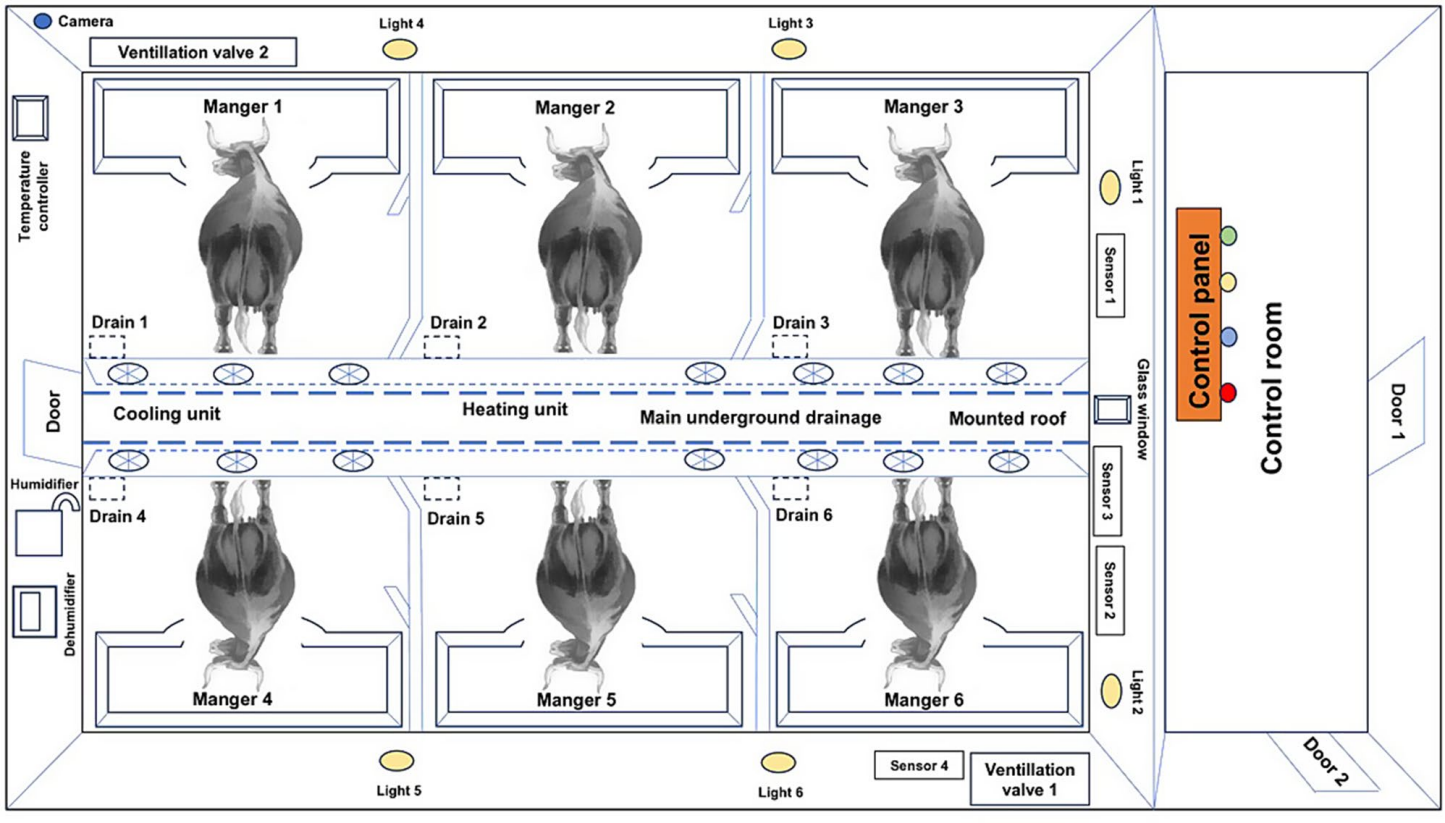
Schematic diagram of the psychrometric chamber used in this study

The guidelines of the Committee for the Control and Supervision of Experiments on Animals (CCSEA), Government of India, were used for experimental procedures as approved by the Institutional Animal Ethics Committee (IAEC), Veterinary University, Mathura (Approval No. IAEC-20/23 dated 30th December 2020). Six Sahiwal zebu calves (age, 8 to 11 months; weight, 120 to 150 kg) were used in this experiment. The animals were reared at the Livestock Farm Complex of Veterinary University, Mathura, and were brought to the experimental facility. They were acclimatized in the antechamber to the psychrometric chamber facility for 10 days before being taken into the psychrometric chamber (Fig. 1). The antechamber has the capacity to accommodate ten animals and is well-ventilated with pucca floors and has a provision of individual feeding, sprinklers, and fans. Two data loggers (Advance Tech India Private Limited, India) were positioned at two corners of the shed to record temperature and humidity. The calves were fed on a total mixed ration (TMR) consisting of wheat straw and a concentrate mixture to meet the predicted nutrient requirements [31]. Deworming of all the experimental heifers was done before the beginning of the experiment by oral administration of Fenbendazole bolus (Intas Pharmaceuticals Pvt. Ltd., India) @ 10 mg/kg body weight, and vaccination against foot and mouth disease, hemorrhagic septicemia and black quarter (Triovac, Indian Immunologicals, India) was done. After every three months, deworming was repeated.

### Experimental design

**Fig. 2:**
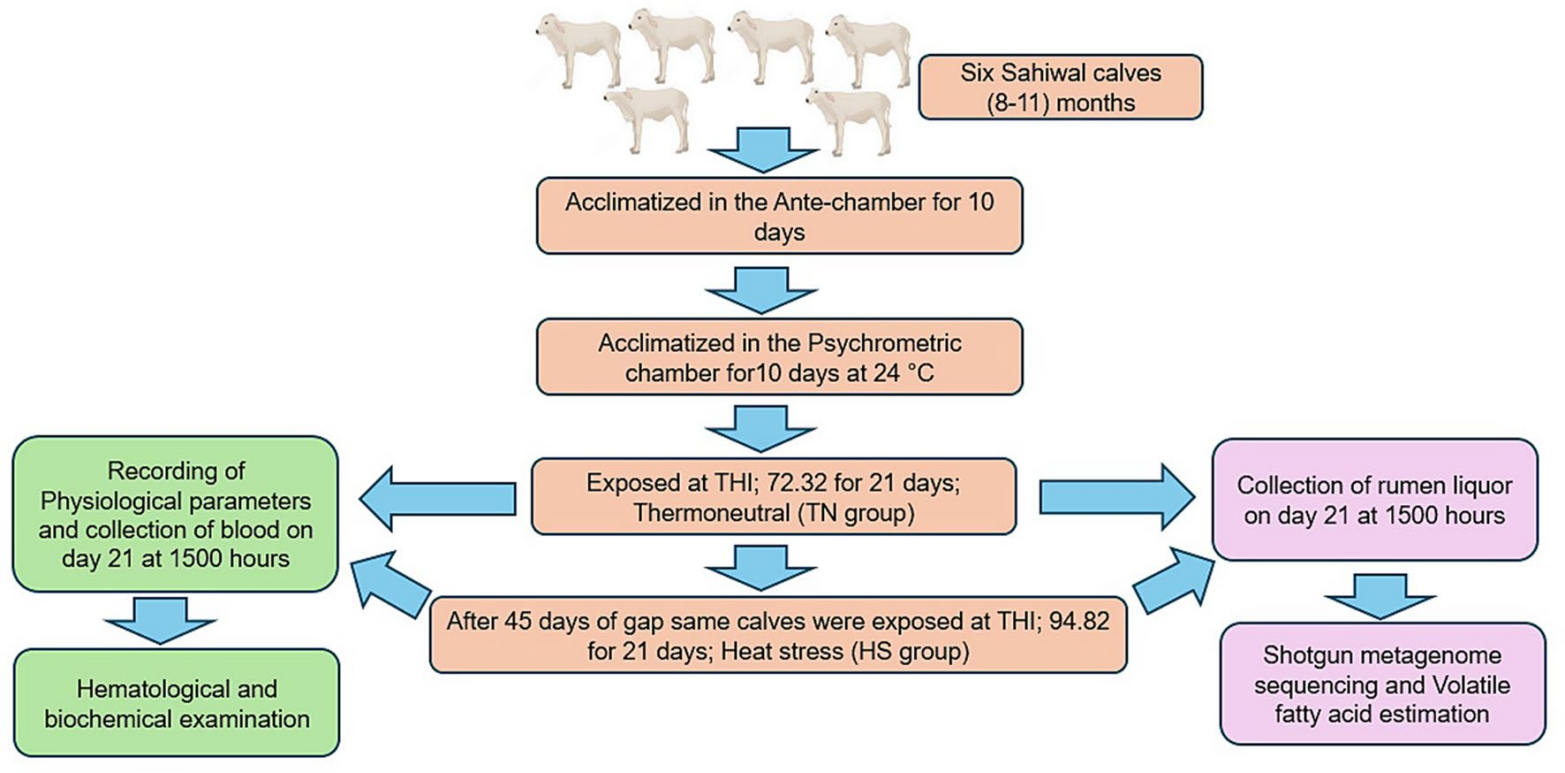
Diagrammatic representation of the experimental design

The experiment was conducted in the psychrometric chamber. Before initiation of the actual experiment, animals were acclimatized in the psychrometric chamber for 10 days at an exposure temperature of 24 °C and a relative humidity of 70% (THI; 72.32). After the initial acclimatization, the Six Sahiwal zebu calves were exposed for six hours every day between 1000 h and 1600 h for 21 days at a TN temperature of 24 °C and relative humidity of 70% (THI; 72.32) in the psychrometric chamber. After a gap of 45 days, the same calves were exposed to the same duration and time at HS temperature of 39 °C and relative humidity of 70% (THI; 94.82) in the psychrometric chamber. The calves were kept in the antechamber from 1600 to 1000 h. During both TN and HS exposures, on 21st day, blood sampling was done, and other physiological parameters were recorded at 1500 h. During both the exposures rectal temperature (RT), respiratory rate (RR), and Pulse rate (PR) were recorded, while blood sampling was done for hematological and biochemical analysis. Rumen liquor was also collected for volatile fatty acid estimation and shotgun metagenome sequencing. The detailed flow diagram of the experimental design is given in Fig. 2.

The temperature humidity index (THI) during both TN and HS in the psychrometric chamber and antechamber was calculated as per the formula of NRC (1971).

$${\rm{THI = (0}}{\rm{.8 \times Tdb) + (RH/100)(Tdb - 14}}{\rm{.4) + 46}}{\rm{.4}}$$

Where, Tdb is the dry bulb temperature and RH is the relative humidity.

### Psychrometric chamber

In the psychrometric chamber, a desired temperature between 5 and 55 °C and relative humidity between 15 and 75% can be maintained within narrow limits (± 1.0). The psychrometric chamber has a dimension of 9.0 m × 4.0 m × 3.0 m with facilities of an individual tie stall and feeders for six adult large animals. It is made up of steel, which is puffed with insulated material to ensure insulation and to keep it airtight. The air passage features one inlet and one outlet valve, both equipped with exhaust fans, positioned diagonally. The chamber is also equipped with temperature, humidity, carbon dioxide level, and light intensity sensors, which are connected with an automation panel located outside the chamber. The required temperature, relative humidity, and carbon dioxide combination is fixed through the automation panel, and it takes 10 to 30 min to achieve the set of climatological variables in the chamber. The chamber has only one main gate for the entry of the animals. The chamber was operated at least half an hour before the animals entered the climatic chamber. The temperature, humidity, and carbon dioxide levels were recorded every five minutes in the automation panel through a data logger.

### Meteorological variables

The meteorological variables of the psychometric chamber and antechamber during the different phases of the experiment are presented in Table 1. The temperature, RH, and THI were recorded following standard methods.

**Table 1:** The climatological variables and Temperature Humidity Index (THI) in the psychrometric chamber and antechamber

Psychrometric Chamber			Antechamber		
Temperature (°C)	RH (%)	THI	Average Temperature (°C)	RH (%)	THI
24	60	72.32	26.45	31.98	75.41
39	60	94.82	31.98	83.6	86.68

### Physiological observations

The PR was recorded by observing the pulsation of the middle coccygeal artery at the base of the tail and expressed as beats per minute. The RR was recorded by observing the flank movement and was expressed as breaths per minute. The RT was recorded by a clinical thermometer (K-life digital thermometer flexible KLF-102) by inserting it into the rectum for at least 1 to 2 min and was measured in degrees Celsius (°C).

### Blood sampling

The blood samples (4–5 mL each) were collected at 1500 h on 10th day of the experiment from the jugular vein by vein puncture and stored in sodium heparin coated vacutainers (BD, Gurugram, India). One mL from each blood sample was subjected to hematological analysis. A part of the blood was centrifuged at 3000 rpm for 30 min to harvest plasma. Blood plasma was stored at ˗20 °C for biochemical examination.

### Hematological analysis

Hematological analysis was done by an automated hematology analyzer (MEK 6550, Nihon Kohden, Japan). White blood cells count (thousand per µL), red blood cells count (millions per µL), haemoglobin (gm/dL), packed cell volume (%), lymphocytes (%), and granulocytes (%) were analyzed in the blood sample.

### Biochemical analysis

Total plasma protein (Catalogue No. 83LS100-60), triglyceride (Catalogue No. 72LS100-60), urea (Catalogue No. 81DP300-72), creatinine (Catalogue No. 85LS200-66) and alanine aminotransferase (ALT; Catalogue No. 76LS200-60), aspartate aminotransferase (AST; Catalogue No. 77LS200-60) and lactate-dehydrogenase (LDH; Catalogue No. 74LS400-60) activity was estimated using commercially available kit (Span Diagnosis Ltd., India) using biochemical analyzer (AutoChem^TM^Xact Pro, Arkray).

### Analysis of cortisol, HSP-70, HSP-90, TNF-α, and IL-6

Bovine-specific ELISA kits supplied by Bioassay Technology Laboratory (Shanghai) were used for estimation of Cortisol (Catalogue No. EO110BO), HSP-70 (Catalogue No. EO226BO), HSP-90 (Catalogue No. EO253BO), TNF-α (Catalogue No. EO019BO), and IL-6 (Catalogue No. EO001BO) as per the manufacturer’s instructions. The intra-assay and inter-assay CV for the estimation of all the hormones were ˂ 8% and ˂ 10%, respectively.

### Collection of rumen liquor

Rumen liquor was collected using the stomach tube method [32]. Rumen liquor was stored in duplicate at -80 °C for further analysis. A part of the rumen liquor was used for VFA estimation, whereas the other part was used for metagenomic studies.

### Analysis of volatile fatty acid (VFA)

After thawing, the rumen liquor was centrifuged at 10,000 x g for 10 min, then the supernatant was collected and processed further for estimation of VFA concentration using gas chromatography (GC) (Agilent Technologies 8890 GC system, Santa Clara, USA). The GC was operated at 50 °C using a separation column (Agilent 19091 S-433UI: T341943H) with a dimension of 30 m x 250 μm x 0.25 μm having a provision of FID detectors. The flow rate in the column was maintained at 1 mL/min whereas the average velocity was 26 cm/sec with a holdup time of 1.92 min. For injector and detector, the temperature was set at 250 °C and 280 °C, respectively.

### DNA isolation and quality check

After thawing of strained rumen liquor samples, 5 mL rumen liquor was used for DNA isolation by the Phenol-Chloroform method [33]. The quality and quantity of the extracted DNA were evaluated using 1.0% gel electrophoresis and NanoDrop spectrophotometer (Thermo Scientific, Wilmington, DE), respectively. The high-quality DNA was then subjected to sequencing.

### Library preparation and shotgun sequencing

High-quality DNA from rumen liquor samples underwent shotgun sequencing. The whole genome sequencing library was prepared using the QIAseq FX DNA Library Kit (QIAGEN, Catalogue No. 180477) following the manufacturer’s instructions. The library was pooled, quantified by qPCR, and sequenced on an SP lane for 151 cycles from both ends (forward and reverse) using the Illumina NovaSeq 6000 platform. The resulting fastq files were generated and demultiplexed using bcl2fastq v2.20 conversion software.

### Reads quality check and subsequent trimming

The obtained reads were subjected to quality check using FastQC v0.12.1 (https://www.bioinformatics.babraham.ac.uk/projects/fastqc/). Based on the read quality report, subsequent trimming was done to improve the read quality. In detail, fastp v0.23.4 [34] was employed to trim the low quality reads (q < 30) with -l 36 and -q 30 parameters.

### Host DNA contamination removal

The DNA obtained from animal guts often contains host contamination, which needs to be removed before downstream analysis to avoid sequence bias and contamination errors. The host contamination was removed using a mapping-based method. Specifically, the *Bos indicus* (Indian zebu cattle) genome was downloaded from the NCBI Genome Database (https://www.ncbi.nlm.nih.gov/datasets/genome/GCF_002263795.3/) and indexed using bowtie2 v2.5.1 [35] with ‘bowtie2-build’ parameter. The generated index files were then used to clean the host contamination, retaining only microbial reads for further analysis.

### Reads taxonomy assignment and classification

The taxonomy assignment for bacteria, fungi, archaea, and protozoa was performed using Kraken2 package v2.0.8 [36]. First, the respective libraries were downloaded using the command ‘kraken2-build --download-library –db library_name’, followed by downloading respective taxonomy with the command’ kraken2-build --download-taxonomy --db taxonomy’. The database was then constructed using the command ‘kraken2-build --build –db database_name’. Taxonomy for the reads was assigned using Kraken2 v2.0.8 [36] with default parameters. For protozoa, rumen specific candidates were taken from NCBI genome and incorporated into the protozoan database. The report file generated by Kraken2 was converted to relative abundance using Bracken v3.1 [37] with default parameters.

### Rumen microbial community functional profiling

The clean reads obtained after host DNA contamination removal were subjected to community functional profiling. In detail, paired end reads were first merged using a ‘cat’ command and then used as input for MetaPhlAn v4.0 [38] to generate taxonomy profile. Functional profiling was then performed using HUMAnN v3.0 [39] with the ChocoPhlAn database v201901b. The resulting ‘pathway_abundance’ files from each sample were merged and normalized using the ‘humann_renorm_table’ command.

### Reads assembly and quality assessment

The read assembly was constructed using metaSPAdes v3.15.5 assembler [40] using default k-mer option. The quality of the assembly file was evaluated using Metaquast v5.2.0 [41]. Based on the quality report, contigs shorter than 1000 bp in length were discarded to obtain high-quality metagenome-assembled genomes (MAGs).

### Bacterial MAG binning and refinement

The final contig file obtained from the metaSPAdes assembler was used to construct bacterial MAGs. We used MetaWRAP v1.2 [42] for binning and subsequent steps. The initial binning was completed using three binning packages: MetaBAT2, MaxBin2, and CONCOCT. After binning, the refinement of bins was performed using the command metawrapbin_refinement with parameters -c 50 -x 5 to retain bins with completeness above 50% and contamination below 5%. The refined bins were then reassembled using the command metawrapreassemble_bins to avoid mismatches and improve bin quality. The final bins obtained were subjected to further downstream analysis.

### Bacterial MAG dereplication and quality assessment

Dereplication of the MAGs was performed using dRep v3.4.5 [43] using default parameters to discard highly similar sequences (> 99%) of MAGs. MAG quality assessment in terms of completeness and contamination was evaluated using CheckM v1.1.3 [44].

### Bacterial MAG classification and phylogenetic tree construction

The classification of bins (MAGs) was evaluated by GTDB-Tk v2.1.1 [45] with the command ‘gtdbtk classify’ using GTDB database [46]. The phylogenetic relationship and clustering were performed using PhyloPhlAn v3.0 package [47], and the results were visualized using the iTOL online platform (https://itol.embl.de/).

### Bacterial MAG annotation, abundance, and average nucleotide identity calculations

The MAG annotation was performed using Prokka v1.14.6 [48] with the following parameters‘--locustag --kingdom --addgenes --evalue –rfam’. The abundance of MAGs was calculated using CoverM package (https://github.com/wwood/CoverM?tab=readme-ov-file). The average nucleotide identity (ANI) was calculated using OrthoANI tool v0.93.1 [49].

### Statistical analysis

The visualization was done with R version v4.0.0 using Vegan [50] and ggplot2 [51] packages. Alpha diversity, specifically the Shannon index, and beta diversity, represented by Principal Coordinates Analysis (PCoA), were calculated at a significance level of *p <* 0.05. Differential species analysis was performed using linear discriminant analysis (LDA) in LEfSe [52], integrated within the MicrobiomeAnalyst platform [53]. The statistical significance of the abundances of phyla and genera between the groups has been tested using the Mann–Whitney method considering p value of 0.05. The physiological responses of the calves during TN and HS period were analysed using paired T test. The significance was set at *p <* 0.05. The Redundancy Analysis (RDA) among microbial taxa and blood parameters was performed in Origin 2021b software package.

## Results

### Alteration of physiological parameters during experimental conditions

A comprehensive set of physiological and biochemical parameters was evaluated, as summarized in Table 2. Changes in these parameters are considered statistically significant at *p* < 0.05 and non‑significant at *p* > 0.05. RT and RR increased significantly during HS, whereas PR showed a slight elevation that did not differ statistically from the TN group. The WBC count, lymphocyte percentage, and granulocyte percentage remained stable across all treatments. In contrast, the RBC count, haemoglobin concentration, and PCV were significantly higher under HS relative to TN condition. Plasma AST activity and triglyceride concentrations declined significantly in response to HS, whereas urea levels and ALT activity did not differ appreciably between groups. Similarly, cortisol and IL‑6 concentrations increased significantly under HS, whereas HSP‑70, HSP‑90, and TNF‑α levels remained unchanged. Rumen VFA concentrations also did not differ between the HS and TN groups (Table 2).

**Table 2:** The physiological, hematological, biochemical parameters and rumen volatile fatty acid during thermoneutral and heat stress exposure in zebu calves

Physiological Parameters				
Parameter	Thermoneutral	Heat Stress	SEM	P Value
Rectal Temperature (° C)	38.579	39.324	0.047	0.001
Respiration rate (Breathes /Minute)	16.667	30.667	2.144	0.001
PR (beats/minute)	66.667	71.333	2.666	0.141
**Hematological Parameters**				
WBC (10^3^/ µL)	11.217	10.367	1.322	0.126
Lymphocyte (%)	72.517	71.900	2.673	0.827
Granulocyte (%)	31.200	27.133	3.301	0.273
Red Blood Cells (Millions/µL)	8.527	7.585	0.381	0.007
Hemoglobin (g/dL)	11.033	9.600	3.804	0.004
PCV	33.067	28.050	1.156	0.005
**Biochemical Parameters**				
AST (IU/mL)	71.879	47.432	5.489	0.007
ALT (IU/mL)	30.117	28.642	3.562	0.696
Triglyceride (mg/dL)	63.537	43.727	5.400	0.014
Urea (mg/dL)	24.597	27.277	3.236	0.445
Cortisol (ng/mL)	5.221	7.831	0.470	0.003
HSP-70 (ng/mL)	11.070	10.389	1.156	0.581
HSP-90 (ng/mL)	74.145	81.951	8.292	0.390
IL-6 (ng/mL)	40.444	48.530	3.804	0.047
TNF-α (ng/mL)	15.617	15.063	1.39	0.324
**Rumen Volatile Fatty Acid**				
TVFA	3.408	3.2300	0.468	0.719
Acetate (%)	73.245	72.642	3.562	0.696
Propionate (%)	19.100	18.920	3.375	0.960
Butyrate (%)	7.663	8.605	3.236	0.771
A: P ratio	4.023	4.120	0.677	0.892

### Sequencing reads quality assessments and related statistics

The raw sequencing data underwent multiple cleaning processes to improve the quality and ensure robust downstream analysis. The read statistics indicated that the average read depth across all samples exceeded 25 million (Table 3). Trimming of adapters and low-quality reads (q < 20) significantly improved the overall read quality, as reflected in the q20 and q30 scores (Table 3). Before processing, the q20 and q30 scores were above 97% and 94%, respectively; however, after processing, these scores improved to over 98% and 95%, respectively.

**Table 3:** Reads statistics and quality assessment of sequencing read before and after processing

Sample ID	Sample groups	Before processing			After processing		
		Total reads	Q20 bases	Q30 bases	Total reads	Q20 bases	Q30 bases
231-Sum_R1 231-Sum_R2	HS	13,005,572 13,005,572	97.57% 97.57%	94.21% 93.39%	12,637,590 12,637,590	98.72% 98.45%	95.77% 94.74%
231-Win_R1 231-Win_R2	TN	16,721,437 16,721,437	97.92% 97.43%	94.39% 94.26%	16,281,134 16,281,134	98.74% 98.41%	95.75% 94.56%
233-Sum_R1 233-Sum_R2	HS	16,387,759 16,387,759	97.27% 97.39%	93.81% 92.99%	15,906,110 15,906,110	98.71% 98.36%	95.74% 94.51%
233-Win_R1 233-Win_R2	TN	14,736,439 14,736,439	97.48% 97.45%	93.91% 92.95%	14,358,137 14,358,137	98.69% 98.32%	95.61% 94.33%
234-Sum_R 234-Sum_R2	HS	18,909,967 18,909,967	97.87% 97.35%	94.52% 92.85%	18,347,363 18,347,363	98.79% 98.28%	96.02% 94.28%
234-Win_R1 234-Win_R2	TN	14,843,147 14,843,147	97.84% 97.08%	94.23% 92.20%	14,406,658 14,406,658	98.72% 98.02%	95.65% 93.60%
238-Sum_R1 238-Sum_R2	HS	37,588,934 37,588,934	97.53% 97.15%	94.23% 92.56%	36,342,895 36,342,895	98.78% 98.18%	95.96% 94.09%
238-Win_R1 238-Win_R2	TN	16,393,243 16,393,243	97.52% 96.98%	93.91% 91.88%	15,904,473 15,904,473	98.70% 97.91%	95.52%
							93.27%
240-Sum_R1 240-Sum_R2	HS	37,535,592 37,535,592	97.71% 97.30%	94.26% 92.72%	36,366,827 36,366,827	98.71% 98.23%	95.78% 94.15%
240-Win_R1 240-Win_R2	TN	18,670,474 18,670,474	97.80% 97.08%	94.16% 92.13%	18,133,706 18,133,706	98.73% 98.02%	95.62% 93.55%
241-Sum_R1 241-Sum_R2	HS	39,853,328 39,853,328	97.95% 97.27%	94.69% 92.66%	38,620,784 38,620,784	98.80% 98.15%	96.03% 93.97%
241-Win_R1 241-Win_R2	TN	14,489,875 14,489,875	97.67% 97.25%	94.02% 92.58%	14,073,424 14,073,424	98.68% 98.16%	95.53% 93.95%

### Bacterial diversity and community analysis

**Fig. 3:**
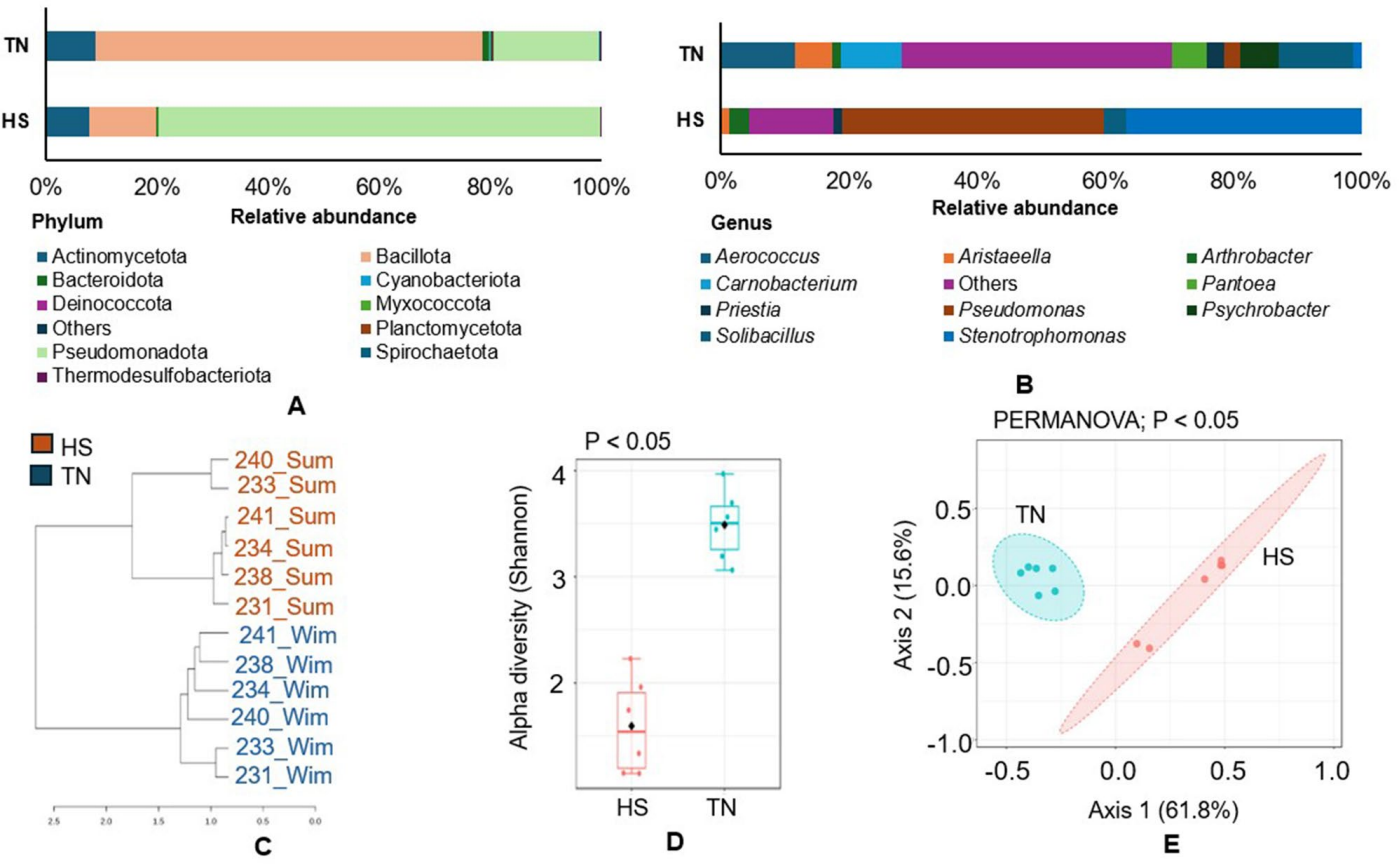
Bacterial community dynamics under different experimental conditions. (**A**) Relative abundance of bacterial taxa at the phylum level. (**B**) Relative abundance of the top 10 bacterial genera. (**C**) Hierarchical clustering of samples based on genus-level composition. (**D**) Alpha diversity measured using the Shannon diversity index. (**E**) Beta diversity analysis illustrates inter-sample variation. Statistical significance was considered at *p* < 0.05

Bacterial community dynamics in the rumen were assessed and are presented in Fig. 3. The results indicate that Pseudomonadota (79.5%) was highly abundant in the HS group, whereas Bacillota (69.8%) was dominant in the TN group (Fig. 3A). Furthermore, statistical tests (*p <* 0.05) revealed significant differences in the abundance of the three phyla between the two groups (Fig. S1A–C). Specifically, Actinomycetota and Myxococcota were significantly more abundant (*p <* 0.05) in the TN group. At the genus level, *Aerococcus* (11.5%) and *Soilbacillus* (11.7%) were highly abundant in most TN group samples (Fig. S1D). In contrast, *Pseudomonas* (40.9%) and *Stenotrophomonas* (36.8%) were dominant in nearly all the HS group samples (Fig. 1B). At the species level, *Pseudomonas simiae* had the highest relative abundance (38.8%) in the HS group, followed by *Stenotrophomonas* sp. BIO128_B (11.3%) and *Stenotrophomonas* sp. LM091 (10.6%). In contrast, the TN group was dominated by *Aerococcus urinaeequi* (11.0%), followed by *Solibacillus silvestris* (9.7%) and *Pantoea agglomerans* (5.0%) (Fig. S1E). A dendrogram based on genus composition clearly separated the samples into two distinct clusters, indicating a strong compositional difference between the groups (Fig. 3C). Additionally, both alpha diversity (Fig. 3D) and beta diversity (Fig. 3E) were significant (*p <* 0.05). The LEfSe analysis further identified the differentially abundant genera between the groups (Fig. S1F). For instance, *Lysobacter* (LDA 3.19) was significantly enriched in the HS group, while a range of bacterial genera, including *Aerococcus* (LDA 4.76), *Carnobacterium* (LDA 4.68), and *Lactobacillus* (LDA 2.0), were enriched in the TN group.

### Fungal diversity and community analysis

**Fig. 4:**
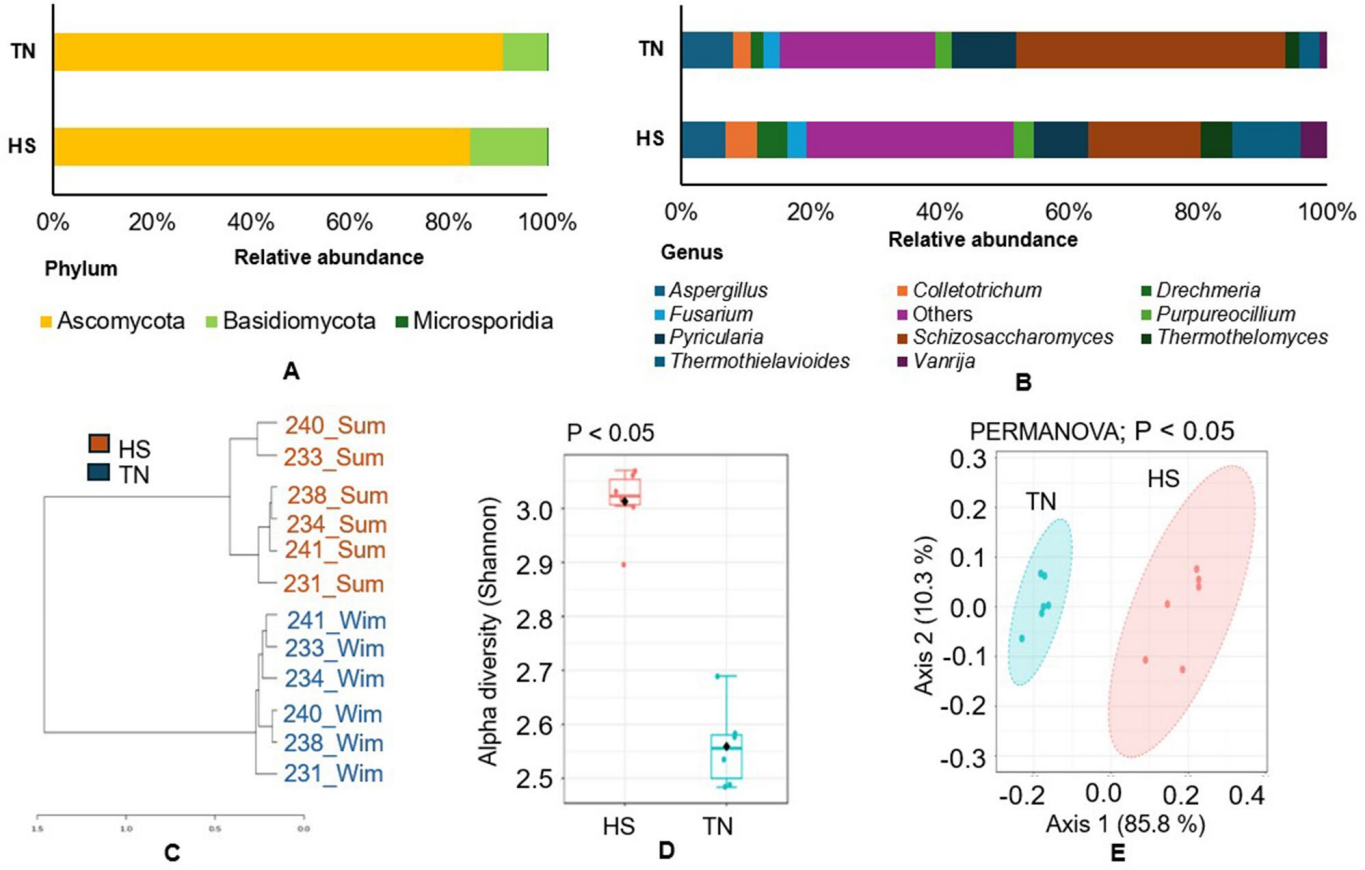
Fungal diversity under thermoneutral (TN) and heat stress (HS) conditions. (**A**) Relative abundance at the phylum level. (**B**) Relative abundance of the top 10 genera under TN and HS. (**C**) Dendrogram based on genus-level distribution across samples. (**D**) Shannon alpha diversity index. (**E**) Beta diversity analysis. Significant differences were determined at *p* < 0.05

The fungal community analysis revealed that the rumen gut was predominantly composed of the phylum Ascomycota (HS: 84.4%, TN: 90.9%), followed by Basidiomycota (HS: 15.4%, TN: 9.0%) in both sample groups (Fig. 4A). At the genus level, *Schizosaccharomyces* was highly abundant in the TN group (41.7%) compared to that in the HS group (17.4%) (Fig. 4B). Conversely, the genus *Thermothielavioides* was more abundant in the HS group (10.4%) compared to the TN group (3.2%). A sample-wise analysis also followed this pattern (Fig. S2A). Species-level analysis revealed that *Schizosaccharomyces pombe* was the most abundant fungal species in both groups, accounting for 15.3% of the community in the HS group and 38.6% in the TN group. In the HS group, the next most abundant species was *Thermothielavioides terrestris* (11.5%), whereas in the TN group, *Pyricularia grisea* (6.8%) had the second highest relative abundance (Fig. S2B). A genus-level dendrogram showed clear segregation between the two groups (Fig. 4C). Alpha diversity, measured using the Shannon index, showed a statistically significant difference (*p <* 0.05) between the groups (Fig. 4D). Similarly, beta diversity was significantly different (Fig. 4E). The LEfSe analysis, considering padj. The P-value was set at *p =* 0.05, and the LDA score was set at ≥ 2.0 to identify the key fungal genera enriched in each group (Fig. S2C). In the HS group, *Thermothielavioides* (LDA 4.56), *Vanrija* (LDA 4.19), and *Thermothelomyces* (LDA 4.16) were significantly enriched. In contrast, *Schizosaccharomyces* (LDA 5.08), *Cutaneotrichosporon* (LDA 3.8), and *Saccharomyces* (LDA 3.58) were enriched in the TN group.

### Archaeal diversity and community analysis

**Fig. 5:**
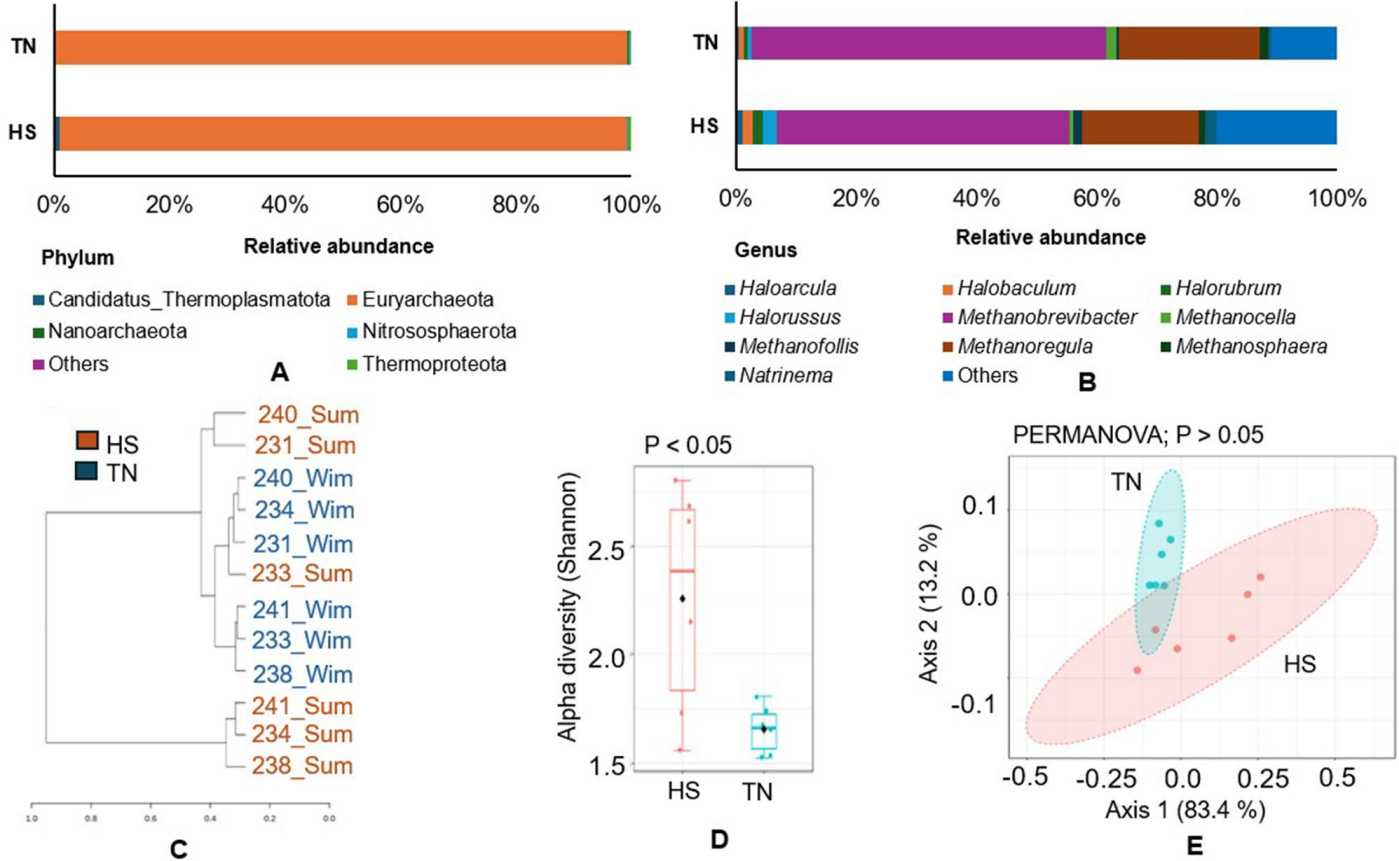
Community dynamics of archaeal populations under HS and TN conditions. (**A**) Relative abundance of archaeal taxa at the phylum level. (**B**) Relative abundance of the top 10 archaeal genera under HS and TN conditions. (**C**) Hierarchical clustering of samples based on genus-level composition. (**D**) Alpha diversity assessed using the Shannon diversity index. (**E**) Beta diversity analysis representing inter-sample variation. Differences were considered statistically significant at *p* < 0.05

The archaeal community was overwhelmingly dominated by a single phylum, Euryarchaeota, in both groups (HS: 98.2%, TN: 99.0%) (Fig. 5A). At the genus level, the abundance patterns were not very distinct between the two groups. In the TN group, *Methanobrevibacter* (59.1%), *Methanoregul*a (23.2%), and *Methanosphaera* (1.5%) were the most dominant genera (Fig. S3A, Fig. 5B). Whereas, in the HS group, *Methanobrevibacter* (48.5%) remained dominant, followed by *Methanoregula* (19.3%) and *Natrinema* (1.8%) (Fig. S3A, Fig. 5B). Species-level profiling showed that multiple *Methanobrevibacter* species were abundant in both groups. In particular, *Methanobrevibacter* sp. YE315 and *Methanobrevibacter millerae* exhibited the highest relative abundance in the HS (20.8% and 13.1%, respectively) and TN (28.3% and 14.5%, respectively) groups (Fig. S3B). Interestingly, in contrast to bacterial and fungal communities, the dendrogram based on the genus-level composition did not distinctly separate the two groups, showing considerable overlap (Fig. 5C). Alpha diversity, measured using the Shannon index, showed a significant difference between the groups (*p <* 0.05) (Fig. 5D). Beta diversity, however, was not significantly different (*p >* 0.05) (Fig. 5E). The LEfSe analysis identified several enriched archaeal genera in each group (Fig. S3C). In the TN group, *Methanocella* (LDA 3.56) and *Methanosarcina* (LDA 3.16) were significantly enriched. Meanwhile, multiple genera, including *Halorussus* (LDA 3.94), *Natrinema* (LDA 3.83), *Halorubrum* (LDA 3.71), and *Methanomassiliicoccus* (LDA 3.61), were enriched in the HS group.

### Protozoa diversity and community analysis

**Fig. 6:**
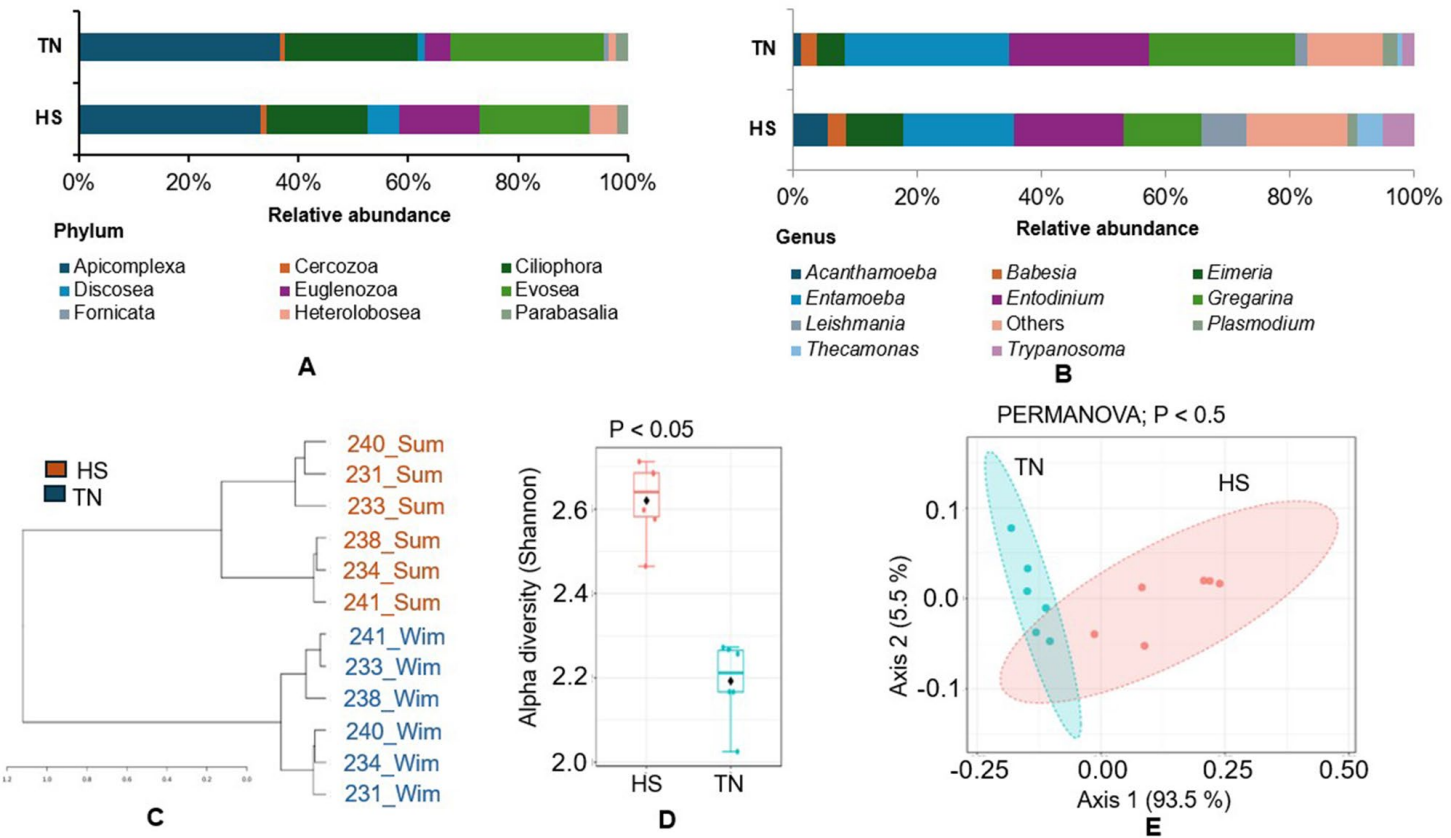
The differential distribution of protozoan community during HS and TN conditions. (**A**) and (**B**) indicate the relative abundance of phylum and genus (top 10), respectively. (**C**) Showing sample dendrogram tree of genus distribution during experimental conditions. (**D**) and (**E**) Represent alpha (Shannon diversity) and beta diversity, respectively. Statistical significance was considered at *p* < 0.05

At the phylum level, Apicomplexa and Ciliophora were the dominant phyla in both groups, representing 32.9% and 18.5% in the HS group, and 36.5% and 24.1% in the TN group, respectively (Fig. 6A). Mann–Whitney test revealed significant differences in the abundance of six phyla: Apicomplexa, Evosea, Heterolobosea, Euglenozoa, Discosea, and Fornicata (Fig. S4 A-F). At the genus level, *Entamoeba* was the most abundant in both groups (HS: 17.8%, TN: 26.3%), followed by *Entodinium* in HS (17.6%) and *Gregarina* in TN (23.3%) (Fig. 6B). A sample-wise analysis showed a similar pattern (Fig. S4G). Species-level profiling revealed that *Gregarina niphandrodes* (HS: 12.4%, TN: 23.2%) and *Entodinium bursa* (HS: 10.5%, TN: 12.5%) were the most dominant species in both groups (Fig. S4H). A dendrogram based on the genus composition formed two distinct clusters (Fig. 6C). Diversity analysis showed a significant difference (*p <* 0.05) in the Shannon diversity index (Fig. 6D), and beta diversity also exhibited a significant difference (*p <* 0.05) (Fig. 6E). LEfSe analysis identified a series of differentially enriched genera in both groups (Fig. S4I). For example, *Leishmania* (LDA 4.43), *Besnoitia* (LDA 3.51), and *Eimeria* (LDA 4.38) were enriched in the HS. In contrast, *Naegleria* (LDA 4.63), *Entamoeba* (LDA 4.63), and *Gregarina* (LDA 4.74) were enriched in the TN condition.

### Bacterial MAGs construction, identification, and abundance

**Fig. 7:**
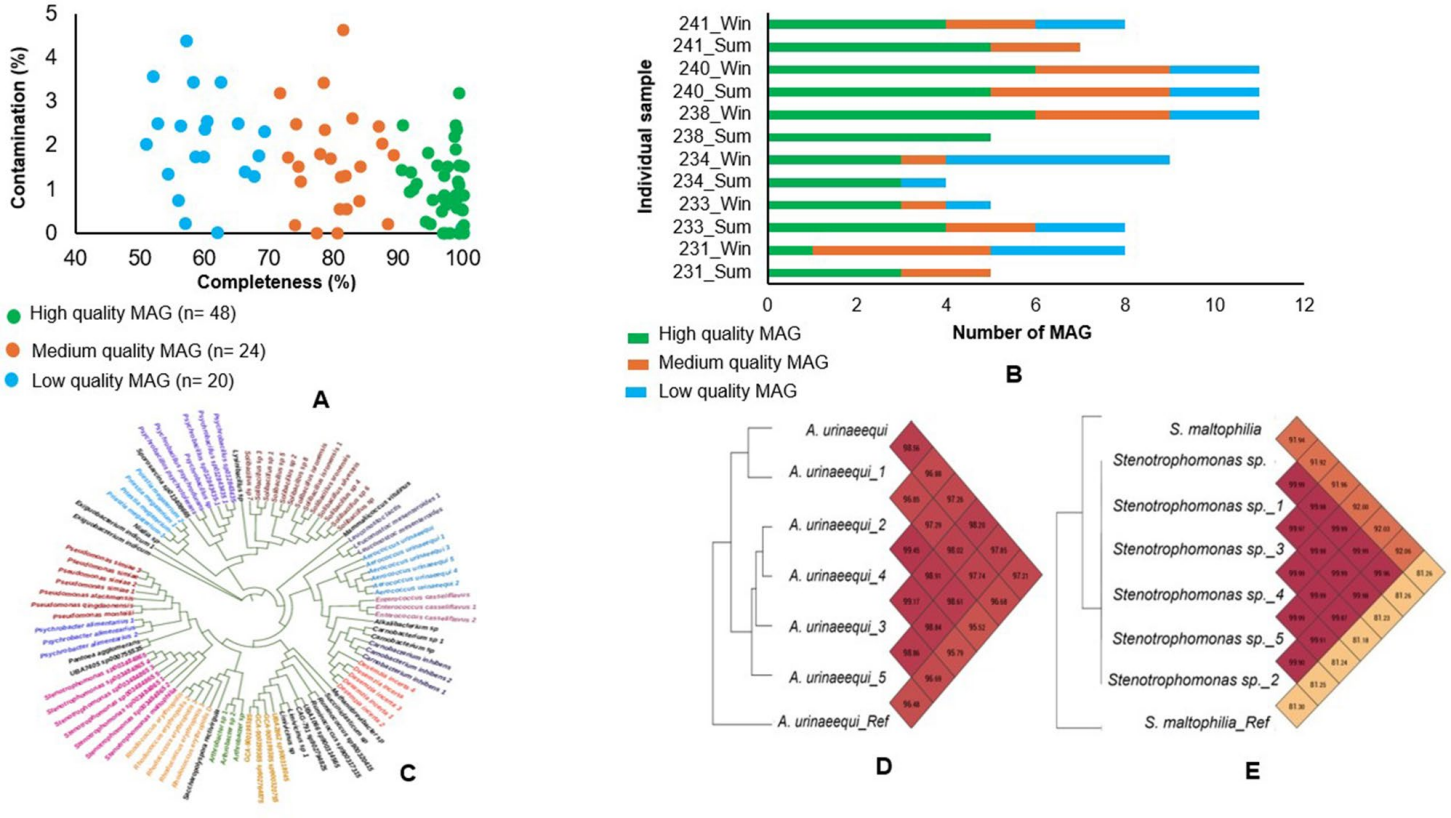
Characteristics of bacterial metagenome-assembled genomes (MAGs) from TN and HS samples. (**A**) Completeness and contamination levels of recovered MAGs. (**B**) Number and quality distribution of MAGs per sample. (**C**) Phylogenetic tree based on whole genome sequences of MAGs. (**D**–**E**) Average nucleotide identity (ANI) analysis of the two most abundant MAGs: Aerococcus and Stenotrophomonas, respectively

In this study, a total of 92 bacterial MAGs were constructed at both genus and species levels from all samples. Among these, 48 were high-quality, 24 were medium-quality, and 20 were low-quality MAGs (Fig. 7A). The quality of the MAGs was evaluated based on completeness and contamination percentages. The results showed that the completeness of the MAGs ranged from 52.7% to 100%, while the contamination levels remained below 5% (Table S1). The highest number of MAGs was observed in samples 240_Win, 240_Sum, and 238_Win, each with 11 MAGs, followed by 234_Win with 9 MAGs (Fig. 7B). A phylogenetic tree was constructed using the MAG genomes, revealing clustering based on genomic similarity (Fig. 7C). *Pseudomonas* genomes were grouped together, while *Stenotrophomonas* and *Aerococcus* genomes formed two distinct clusters. Relative abundance analysis based on genome mapping methods showed that *Pseudomonas simiae* and *Stenotrophomonas* sp. 003484865 were the most abundant species in 231_Sum, accounting for 26.06% and 26.07% of the community, respectively (Table S2), respectively. In contrast, *Desemzia incerta* (52.9%) and *Aerococcus urinaeequi* (7.72%) were dominant in 231_Win (Table S2). The occurrence of MAGs of *Stenotrophomonas* sp. were more frequent in the HS group samples, whereas *A. urinaeequi* MAGs were commonly found in the TN group samples. The average nucleotide identity (ANI) analysis showed that all *A. urinaeequi* MAGs exhibited high similarity, with values above 95% (Fig. 7D), while MAGs of *Stenotrophomonas* sp (Fig. 7E) also displayed strong genomic closeness, exceeding 91%. The annotations of all MAGs are presented in Table S3.

### Pathways enrichment and differential abundance between groups

**Fig. 8:**
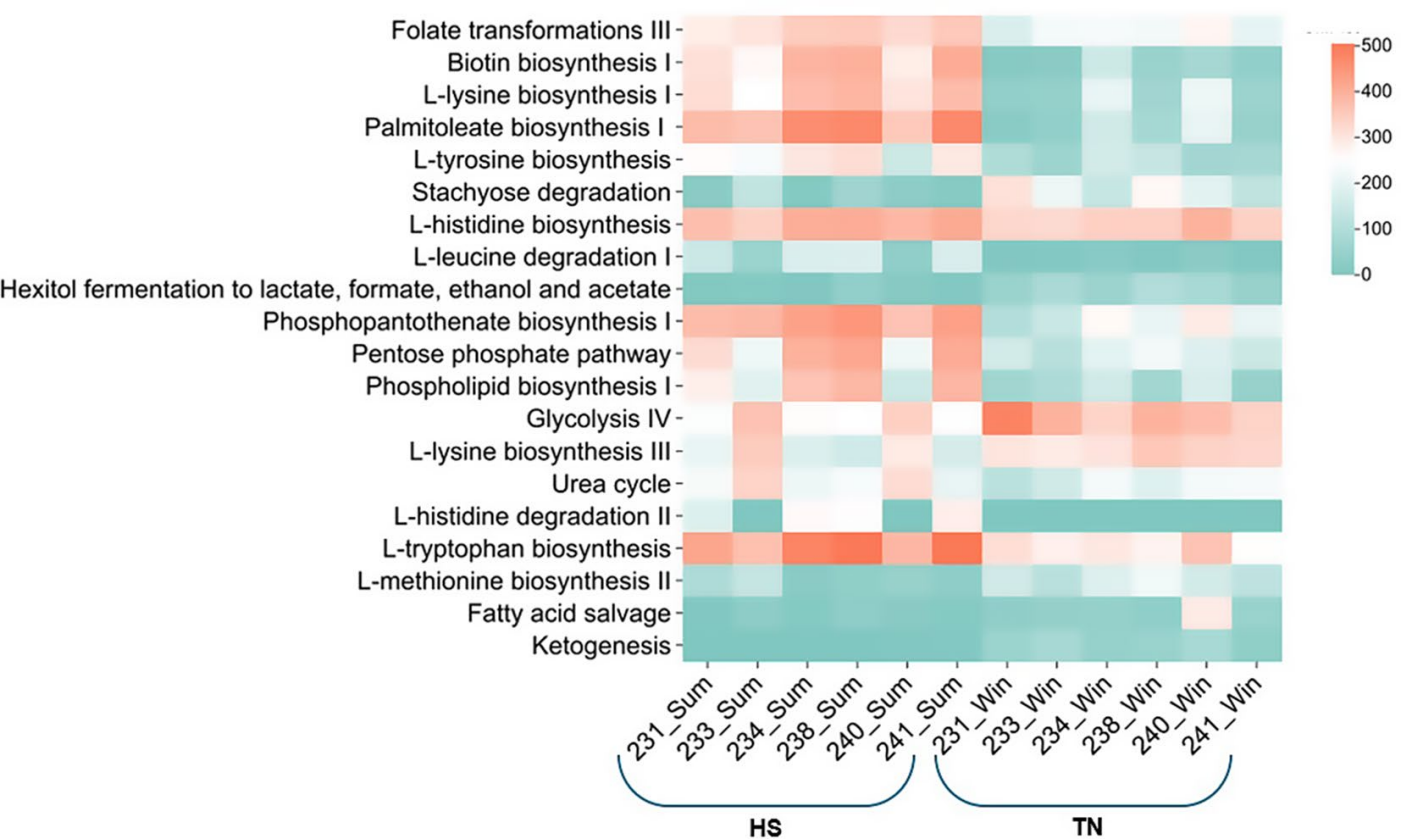
The distribution and enrichment of different metabolic pathways during HS and TN experimental conditions

To examine the impact of temperature on metabolic pathways, we analyzed the shotgun metagenomic data (Fig. 8). The results revealed differential abundance across various pathways. Notably, the folate transformations III and biotin biosynthesis I pathways were significantly enriched (*p* < 0.05) in most HS group samples, with abundance levels ranging from 286.28 to 355.39 CPM and 264.95 to 410.43 CPM, respectively (Fig. 8). In addition, several amino acid biosynthesis pathways, including L-lysine biosynthesis I, L-tryptophan biosynthesis, and L-tyrosine biosynthesis, were significantly elevated (*p* < 0.05) in the HS group compared those to in the TN group. The L-histidine biosynthesis pathway also exhibited higher (but statistically non-significant) abundance in the HS group. In contrast, the glycolysis IV pathway was significantly more abundant (*p* < 0.05) in the TN group. Similarly, the L-lysine biosynthesis III pathway was more abundant in the TN group than in the HS group; however, this difference was not statistically significant.

### Correlation between microbiome and physiological parameters

**Fig. 9:**
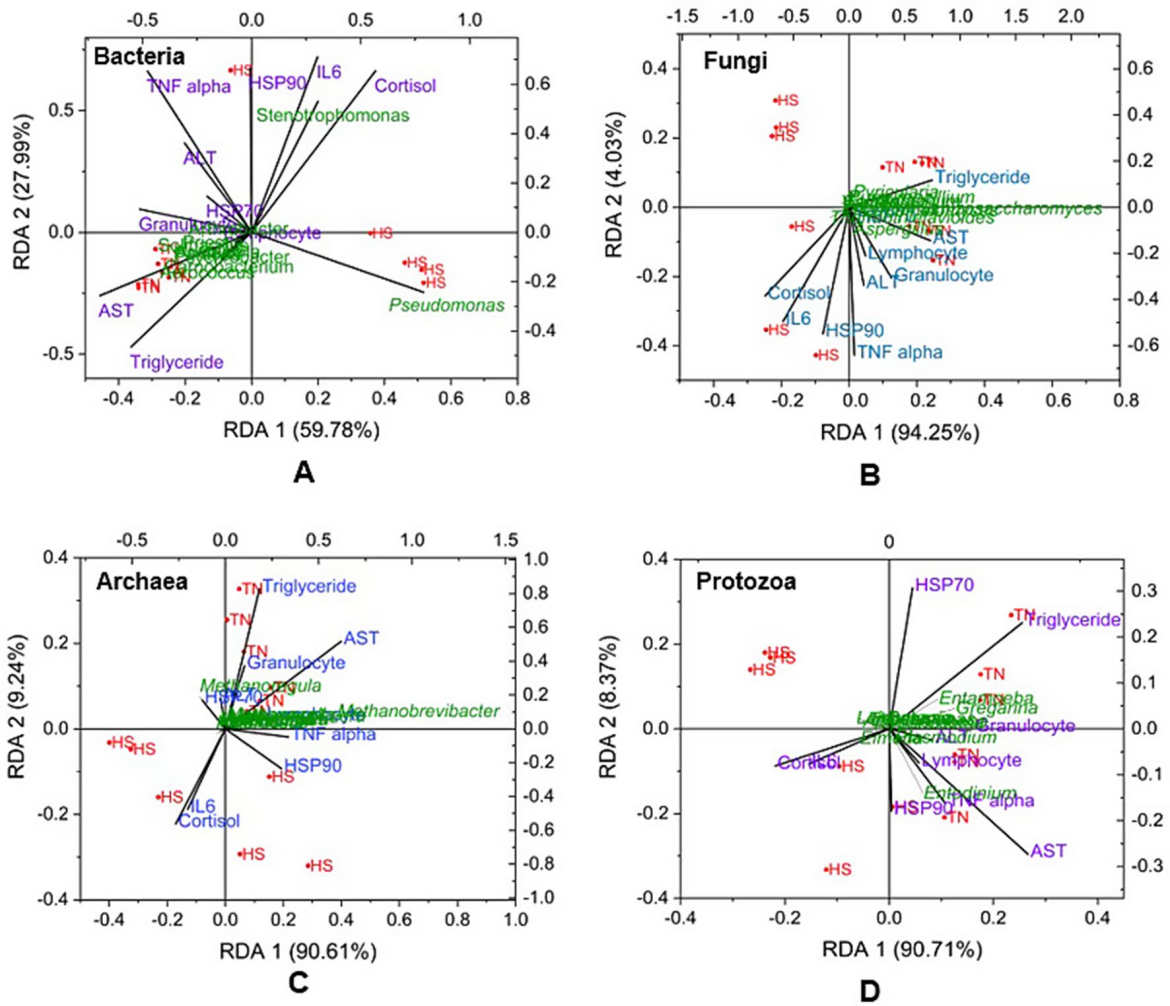
Redundancy analysis (RDA) of microbiome-physiology associations under TN and HS conditions. (**A**) RDA plot illustrating correlations between bacterial genera and host physiological parameters. (**B**) RDA plot showing associations between fungal taxa and physiological markers. (**C**) Correlation between archaeal communities and physiological parameters. (**D**) Correlation between protozoal taxa and host physiological responses

RDA analysis showed a strong correlation between microbial abundance and physiological parameters under HS and TN conditions (Fig. 9). The bacterial community variation was explained by 87.77%, with *Stenotrophomonas* showing a strong positive correlation with stress markers like HSP-90, IL-6, and cortisol, while *Aerococcus*, *Arthrobacter*, and *Carnobacterium* correlated with AST and triglyceride levels (Fig. 9A). *Pseudomonas* was highly abundant in the HS group but negatively correlated with all physiological parameters (Fig. 9A). The fungal community variation was primarily explained by RDA Axis 1 (94.25%), with HS samples linked to stress markers (cortisol, IL-6, HSP-90, and TNF-α) and TN samples associated with metabolic and immune markers (triglycerides, AST, and lymphocytes) (Fig. 9B). *Aspergillus* correlated positively with lymphocytes, ALT, and granulocytes, while most fungal taxa had minimal influence on physiological parameters (Fig. 9B). The archaeal variation (97.01%) showed *that Methanoregula* positively correlated with granulocytes, AST, HSP-70, and triglycerides, whereas *Methanobrevibacter* (more abundant in TN) correlated with ALT and lymphocytes (Fig. 9C). Among protozoa, *Entamoeba* and *Gregarina* were positively correlated with HSP-70 and triglyceride levels, whereas *Entodinium* exhibited a positive correlation with AST, TNF alpha, HSP-90, and lymphocyte levels (Fig. 9D).

## Discussion

Ruminants harbor a complex and dynamic consortium of microorganisms in their rumen, collectively referred to as the rumen microbiome. This microbial ecosystem maintains a symbiotic relationship with the host and plays essential roles in nutrient metabolism, immune modulation, and overall physiological homeostasis. However, environmental stressors, particularly HS, pose substantial challenges to the host–microbiome interface [54]. HS impairs thermoregulatory mechanisms and alters key physiological functions, such as feed intake, metabolic efficiency, and immune responses in ruminants [7]. Recent evidence indicates that HS also induces significant shifts in the composition, diversity, and metabolic activity of the gut microbiome, thereby increasing the susceptibility of animals to health disorders and compromising their productivity [30, 54]. The present study investigated HS–induced alterations in the rumen microbiome and their interactions with host physiological responses in zebu cattle, which are recognized for their inherent thermotolerance. Understanding these interactions is critical for developing targeted strategies to enhance resilience, sustain productivity, and promote animal welfare in the face of increasing climatic variability.

### High heat stress significantly alters physiological, hematological, and biochemical parameters in zebu calves

In the present study, the THI values during TN and HS exposure were 72.32 and 94.82, respectively. At a THI of 72.32, the zebu animals experience no stress, whereas at a THI of 94.82, they suffer from extreme HS [7]. The physiological responses observed at THI 94.82 showed significant deviations from those under TN condition, indicating that the animals were under HS. Rectal temperature (RT) is one of the most reliable indicators of HS [2]. In the present study, RT increased by approximately 0.8 °C, suggesting an elevated heat load and overall body temperature [55]. In heat-tolerant zebu cattle, RT typically increases only modestly under high THI, often by 0.3–0.4 °C [56]. In the present study, calves exhibited an RT increase of 0.8 °C, which is above the expected physiological response for zebu breeds and therefore represents a clear heat-stress condition. Jose et al. (2022) reported a 1.6 °C increase in RT during severe HS in zebu calves [57]. The results obtained under HS conditions suggest that physiological heat-loss mechanisms are insufficient to maintain an optimal body temperature.

In this study, respiratory rate (RR) nearly doubled during HS exposure, a finding consistent with the results reported by Jose et al. (2022) in zebu calves exposed to severe HS [57]. An increase in hemogram values was also observed, likely due to hemoconcentration resulting from HS, as reported in previous studies [6]. However, increased hemogram values may also arise from splenic contraction and blood volume redistribution, which transiently elevate RBC, Hb, and PCV values [56]. No change was observed in the percentage of granulocytes and lymphocytes, which may vary among livestock species under HS [58]. Changes in plasma AST activity and triglyceride levels indicate altered liver function and fat metabolism during HS, consistent with previous reports [4]. Interestingly, only a minimal increase in plasma HSP-70 and HSP-90 levels was observed in zebu calves, unlike the significant elevations reported in other studies on cattle [4, 59].

Cortisol, a key marker of HS [11, 60], was found to be elevated during HS exposure in this study. Cortisol is known to influence bone marrow cell dynamics, metabolism, and immunity [61]. While TNF-α and IL-6 are often reported to vary under HS [62], only IL-6 was found to increase in the present study, in parallel with cortisol. Chen et al. (2018) similarly reported elevations in both TNF-α and IL-6 under HS in cattle [11]. These results indicate that HS compromises the immune system of zebu calves. Rumen liquor analysis showed no change in volatile fatty acid (VFA) concentrations in the heat-stressed group, suggesting that the rumen microbial ecosystem was not significantly altered. The impact of HS on VFA production varies considerably among livestock species [8].

### Heat stress significantly alters the rumen microbiome in zebu calves

The microbiome in the animal gut, including the rumen, plays a crucial role in maintaining host digestion, metabolism, and physiological function. The microbiome comprises bacteria, fungi, archaea, viruses, and protozoa in specific environments. In this study, we considered four major domains of microbiota to assess the impact of temperature on rumen physiology and health.

#### Heat stress reduced the bacterial community diversity

The bacterial community dynamics showed a shift between two dominant phyla: Pseudomonadota (formerly Proteobacteria) and Bacillota (formerly Firmicutes) [63] in response to HS. Both are major phyla in the rumen microbiome [64], comprising Gram-positive and Gram-negative bacteria, respectively. The higher abundance of Bacillota in the rumen is shown to be beneficial, as members of this phylum help break down plant material, including fibers, into VFAs and other energy-yielding compounds essential for the host [65], and its decreased abundance indicates that fiber-degrading capability may be decreased during HS. Conversely, an increase in Pseudomonadota abundance may pose health risks, as some members are opportunistic pathogens [66]. The elevated Pseudomonadota levels under HS suggest gut dysbiosis, impacting rumen’s health and animal productivity. *Pseudomonas*, a Gram-negative bacterium, comprises several species that are known to cause a wide range of diseases, including bovine laminitis [67] and mastitis [68]. Its presence in the rumen has also been previously reported [69]. Interestingly, species-level analysis indicated an enrichment of *Pseudomonas simiae* in the HS group. However, this species is not commonly found in the rumen. Several strains of *P*. *simiae* have been reported in soil-associated environments and are recognized as beneficial to hosts [70]. Additionally, this bacterium has been confirmed in bovine milk [71]. Therefore, its detection in cattle requires further validation, such as isolation, species-specific PCR, or whole-genome sequencing, to verify its true association with the host. Similarly, several species of *Stenotrophomonas*, like *Stenotrophomonas* sp. BIO128_B and *Stenotrophomonas* sp. LM091 were also very high in the HS group. A previous study reported an increase in *Stenotrophomonas* abundance in heat-stressed buffalo heifers [30], and its high abundance has been linked to mastitis in dairy cattle and detected in milk [72]. Therefore, the high abundance of *Pseudomonas* and *Stenotrophomonas* in the HS group may negatively impact the rumen gut microbiota. Furthermore, our results showed a relatively higher *Solibacillus* abundance under TN condition than HS, although contrary findings have been reported in dairy cattle during summer [73]. The presence of *Solibacillus* in the rumen has been reported in feed fermentation [74], and its abundance may correlate with reduced dietary intake, a common occurrence during transportation [75]. However, *Solibacillus* may be a part of the normal rumen microbial community, and its abundance has been linked to dietary changes [76]. Research on *Solibacillus* remains limited, and its functional role within the rumen ecosystem has not yet been fully elucidated. Therefore, the reason for its high abundance in the TN group remains unclear, and further investigation is required. Species-level profiling revealed a high abundance of *Aerococcus urinaeequi* and *Pantoea agglomerans* in the TN group. *Aerococcus urinaeequi* is a Gram-positive facultative anaerobe that has been isolated from various environmental sources and livestock [77], although reports of its association with cattle remain limited. In contrast, *Pantoea agglomerans* is a Gram-negative, plant-associated bacterium frequently found in soil [78], and animal farm environments [79], but it is not considered a typical rumen inhabitant. Therefore, the presence of both species in the rumen is likely due to transmission from feed or cattle-associated environmental sources rather than stable colonization by these species.

Alpha diversity analysis showed a higher Shannon index under TN than under HS condition, indicating greater species richness during TN condition. Under HS, *Pseudomonas* and *Stenotrophomonas* dominance reduced rumen microbial diversity. Simultaneously, beta diversity analysis revealed a significant shift in the bacterial community structure under HS. LEfSe analysis highlighted the significant taxa associated with each condition. Several bacterial genera, including *Aerococcus*, *Carnobacterium*, *Lactobacillus*, and *Aristaeella*, were enriched under the TN condition. In a previous study, Wang et al. showed that *Aerococcus* level*s* decreased in HS dairy cows but increased after herbal supplementation [80]. *Carnobacterium* abundance was shown to increase during winter in Holstein and Jersey steers [81], which is consistent with our findings. However, *Lactobacillus* has been found to be enriched in the HS condition in livestock in some cases [26, 30], which contradicts our results. Some Gram-positive bacteria (e.g., *Aerococcus*, *Carnobacterium*, and *Lactobacillus*), found in our study, were also identified in the healthy rumen gut and milk [82, 83]; however, their role in host performance remains unexplored. *Aristaeella*, found to be enriched under TN condition, facilitates the digestion of plant materials like xylan and pectin [84]. Under HS, *Lysobacter* was enriched - a Gram-negative bacterium that survives extreme conditions [85] and may influence pyruvate and butanoate metabolism [86]. Overall, the bacterial diversity analysis revealed a distinct microbial community shift between the TN and HS conditions, indicating that HS may significantly alter the normal gut bacterial population.

#### Fungal community shift under heat stress

The rumen hosts a diverse fungal community that plays a vital role in breaking down fibrous plant material, aiding digestion in ruminants [87]. Our results indicated that the fungal phyla Ascomycota and Basidiomycota were highly abundant under both TN and HS conditions in the present study. These fungi are known to produce mycelia that degrade plant fibers, thereby enhancing digestive efficiency [88]. Ascomycota includes species such as baker’s yeast and penicillin-producing molds, whereas Basidiomycota also contributes to fiber breakdown [87, 89]. At the genus level, *Schizosaccharomyces* was dominant in both groups. *Schizosaccharomyces*, a fission yeast found in fruits and honey, has been observed in gayals, but its rumen function remains unclear [90, 91]. Several species of *Schizosaccharomyces* have been reported as fiber degraders. For example, *S. pombe* has been identified as a lignocellulose-degrading yeast [92] that may contribute to ruminal fermentation end products [93]. In our study, *S. pombe* showed markedly higher abundance in the TN group; however, VFA concentrations did not differ significantly between the HS and TN groups (*p >* 0.05). This observation aligns with the current understanding of rumen ecology, where bacteria are the primary producers of VFAs, while fungi and yeasts, such as *Candida*, *Saccharomyces*, and *Schizosaccharomyces*, mainly participate in fiber degradation and indirectly support bacterial fermentation. Therefore, an increase in yeast abundance alone may not substantially influence the abundance or activity of key VFA-producing bacterial genera, including *Prevotella*, *Butyrivibrio*, and *Ruminococcus* [94]. In contrast, *Thermothielavioides* (particularly *T. terrestris*), a thermophilic fungus, is known for lignocellulose degradation and was more abundant under HS, suggesting an adaptive response to maintain fiber digestion during heat stress [95, 96]. The increased abundance of *Thermothielavioides* during HS seems to suggest an adaptive strategy of the rumen ecosystem to compensate for the altered rumen physiology. However, its presence in the rumen and its relationship with temperature fluctuations remain largely unexplored. Diversity analyses revealed that fungal species richness and community composition significantly differed between the TN and HS groups. The HS group showed higher alpha (species richness) and beta (community structure differences) diversity, indicating a more diverse fungal community under stress. This aligns with previous findings in yaks, where higher microbial diversity was linked to environmental adaptation [97]. LEfSe analysis identified specific fungi that were enriched under each condition. Under TN condition, *Candida* and *Saccharomyces* were more prevalent, both of which are known to aid in fiber digestion [98, 99]. *Eremothecium*, *Naumovozyma*, and *Cutaneotrichosporon* were enriched under HS *Eremothecium*, typically not a rumen resident, produces riboflavin, possibly meeting increased vitamin needs during HS. *Naumovozyma*, related to *Saccharomyces* [100], may contribute to fermentation, although its role in the rumen is not fully understood [97]. *Cutaneous trichosporon*, a lipase-producing yeast [101], may enhance fat digestion under HS. Overall, the enrichment of specific fungi during HS suggests an adaptive shift in the rumen microbiome to maintain the digestive efficiency. However, further research is needed to clarify the functional roles of these fungi in nutrient metabolism and microbial adaptation to HS.

#### Shift in archaeal community

The archaeal community in the rumen plays a critical role in methanogenesis by converting hydrogen and carbon dioxide into methane. While essential for maintaining fermentation and hydrogen balance, this process results in a 2–12% loss of dietary energy and contributes to global warming. In our study, Euryarchaeota was the dominant phylum, and *Methanobrevibacter* was the predominant genus under both TN and HS conditions, with their abundance remaining stable during the HS. However, at the species level, *Methanobrevibacter* sp. YE315 and *Methanobrevibacter millerae* were enriched in both groups of animals. All species within the *Methanobrevibacter* genus are known methane producers [102] and play a crucial role in rumen fermentation by scavenging hydrogen generated during the microbial degradation of feed. Therefore, the higher abundance of these methanogenic archaeal species in the TN group may enhance fermentation efficiency by maintaining the hydrogen balance in the rumen. Our findings align with previous reports showing that archaeal populations remain stable under acute HS [21, 103], suggesting that archaea may possess thermotolerance or occupy niches less susceptible to thermal shifts. However, the literature shows varying patterns: Yadav et al. (2022) observed decreased *Methanobrevibacter* abundance in heat-stressed buffalo [30], whereas Sales et al. (2021) reported an increase in beef cattle [23]. Although the dominant taxa remained unchanged, alpha diversity indices and LEfSe analysis showed that HS altered archaeal diversity. Under TN condition, *Methanocella*, which uses only hydrogen for methanogenesis, was enriched. During HS, *Methanosarcina*, capable of utilizing multiple substrates, was enriched, indicating a shift toward versatile methanogens. Halophilic archaea were enriched under HS, suggesting increased rumen osmolarity. Additionally, *Methanomassiliicoccus*, a hydrogen-dependent methylotrophic methanogen, was enriched during HS, possibly reflecting increased methylated substrates. These findings indicate that while core methanogens remain stable, HS induces shifts in archaeal community composition, potentially affecting methane production and rumen fermentation efficiency.

#### Heat stress induced shift in protozoan community with the enrichment of potentially pathogenic species

Rumen protozoa are essential microbiome members, comprising 20–50% of the total microbial biomass [104]. Their functional roles remain largely unexplored, although defaunation negatively affects organic matter digestion [105]. Our results show a high relative abundance of Apicomplexa, Evosea, and Ciliophora under both TN and HS conditions. Ciliophora has been linked to elevated methane production in the rumen [106]. However, members of this phylum may engage in symbiotic relationships with the host [107]. Previous studies have shown that various rumen protozoa belonging to the phylum Ciliophora possess the potential to produce fibrolytic enzymes and may contribute to rumen metabolic processes [108]. Therefore, the higher abundance of Ciliophora observed in the TN group may suggest a potentially beneficial association, although further functional validation is required to confirm this effect. Furthermore, Euglenozoa exhibited higher abundance in the HS group, indicating potential health risks. Evosea consists of heterotrophic flagellates that have been previously reported in the rumen, although their exact role in digestion remains unclear [109]. Genus- and species-level profiling revealed a higher relative abundance of several protozoan taxa in the TN group, including *Gregarina* (*Gregarina niphandrodes*) and *Entodinium* (*Entodinium bursa*). Multiple members of the genus *Entodinium* are well-documented rumen inhabitants [110], including *E. bursa* [111], which has been proposed as a potential contributor to fiber degradation and amino acid metabolism in the rumen [111]. In addition, *Entodinium caudatum*, one of the most predominant and functionally important rumen ciliate species, was detected in both groups and is known to play a key role in rumen metabolic processes [112]. In contrast, *Gregarina niphandrodes* is an apicomplexan parasite primarily reported in invertebrate hosts [113], and its detection in rumen samples is unlikely to reflect actual colonization. Instead, this observation may result from limitations in current protozoan reference databases. Interestingly, the TN group showed higher relative abundances of *Entamoeba dispar* and *Entamoeba histolytica* than the HS group. Associations between *Entameba* species and vertebrate hosts, including cattle, have been previously reported [114]. *E. histolytica* is generally recognized as a parasitic species associated with diarrheal disease, while *E. dispar* is considered a non-pathogenic commensal [115]. However, the ecological roles and functional significance of *Entamoeba* spp. within the rumen environment remain unclear and need further investigation.

Alpha diversity showed higher species richness in the HS group, while beta diversity revealed significant (*p <* 0.05) differences between groups. Previous research found lower Shannon diversity in winter than in summer [81]. Gut microbiome diversity, including protozoan diversity, is influenced by multiple factors, such as seasonal temperature, diet composition, and host physiology [97]. Previous investigations of the rumen microbiome have reported lower Shannon diversity in winter than in summer [116], which is consistent with our observations. These findings suggest that seasonal temperatures may indirectly shape gut microbial communities by altering diet quality, thereby contributing to seasonal shifts in microbial diversity.

LEfSe analysis indicated the differential enrichment of several protozoan taxa with known parasitic potential between the two groups. Specifically, *Naegleria* and *Entamoeba* were enriched in the TN group, whereas *Leishmania*, *Besnoitia*, and *Eimeria* were enriched in the HS group. These protozoan genera are generally recognized as pathogenic in vertebrate hosts and have been associated with severe disease [117–120]. Interestingly, previous studies have also reported the detection of several of these taxa in the rumen, including *Leishmania* [121, 122], *Entamoeba* [123], and *Besnoitia* [124], which is consistent with our findings. However, these protozoa are not considered stable residents of the rumen ecosystem [125]. Therefore, their detection in our sample might be due to environmental exposure rather than active colonization in the gut or a direct effect of temperature.

### High-quality bacterial MAGs revealed distinct rumen microbial signatures under thermoneutral and heat stress conditions

The recovery of MAGs from shotgun metagenomic data is a critical step in functional metagenomics, as it enables detailed insights into host–microbe interactions, microbial community functions, and the discovery of novel microbial taxa [126]. In this study, we successfully reconstructed a substantial number of bacterial metagenome-assembled genomes (MAGs; *n* = 92) from the analyzed samples and assessed their qualities. According to the Minimum Information about a Metagenome-Assembled Genome **(**MIMAG**)** standards for bacteria and archaea, the most essential criteria for MAG quality are genome completeness and contamination [127]. High-quality MAGs are typically defined as having > 90% completeness and < 5% contamination. Interestingly, our analysis revealed a high abundance and distribution of *Stenotrophomonas* sp. and *Actinomyces urinaeequi* MAGs in the HS and TN samples, respectively. The enrichment of *Stenotrophomonas* in heat-stressed (HS) samples is intriguing; although the underlying mechanism is unclear, previous studies have associated this genus with the progression of mastitis in dairy cows [72]. In contrast, *A. urinaeequi* and other beneficial microbial candidates have been reported to inhibit *Escherichia coli* colonization in the rumen, thereby contributing to improved gut health in cattle [82]. These findings suggest that the TN condition may support a more beneficial and health-associated microbial community compared to heat-stressed condition.

### Enrichment and differential abundance of key metabolic pathways reveal rumen microbial metabolic plasticity as a key factor in host resilience to thermal stress

Folate is an essential nutrient for the growth of rumen cellulolytic bacteria and plays a critical role in milk synthesis and antioxidant defense [128]. Additionally, folic acid is vital for DNA synthesis and methionine metabolism, further underscoring its importance in cellular and microbial functions [129]. In the present study, the enrichment of folate transformation III, biotin biosynthesis, and amino acid biosynthesis pathways in the rumen microbiome during HS suggests a microbial adaptive response aimed at maintaining functional stability and homeostasis within the rumen ecosystem. This metabolic adaptation is reflected in the minimal changes observed in volatile fatty acid (VFA) production, even under HS condition. In contrast, several studies conducted in *Bos taurus* cattle under HS have reported both a reduction in total VFA concentration and alterations in VFA composition, likely due to the disrupted rumen microbial activity. However, in the current study, as well as in earlier observations in buffaloes [30], no significant changes in VFA concentration were detected during HS. This stability in VFA levels may indicate greater heat resilience, not only of the host species (e.g., the zebu) but also of their rumen microbiome. The ability of the microbial community to upregulate biosynthetic and metabolic pathways associated with nutrient synthesis and oxidative protection likely contributes to the preservation of rumen fermentation efficiency, even under thermal stress. These findings highlight the importance of microbial functional plasticity as a key factor in host adaptation to environmental stressors.

### Elevated biomarkers of inflammation and systemic stress strongly correlated with the shifts in the rumen microbiome

Heat stress (HS) in livestock is characterized by a series of physiological and molecular responses, with HSP-90, IL-6, and cortisol being widely recognized as key biomarkers reflecting cellular stress, systemic inflammation, and systemic stress, respectively [3, 7]. However, the role of the rumen microbiome in modulating these biomarkers during HS remains inadequately understood. In the present study, a notable increase in *Stenotrophomonas* abundance was observed during HS, which showed a strong positive correlation with levels of HSP-90, IL-6, and cortisol. This is consistent with earlier findings in buffaloes [30]. *Stenotrophomonas* has also been implicated in sub-acute ruminal acidosis (SARA), where it was found to proliferate in conjunction with elevated levels of lipopolysaccharides (LPS) in the rumen fluid and bloodstream [72]. SARA has been linked to increased concentrations of IL-6 and cortisol [130], indicating a potential commonality in the pathophysiological mechanisms of SARA and HS. Moreover, HS has been shown to increase endotoxin levels in cattle [131], which may compromise epithelial integrity and contribute to systemic inflammation. Therefore, the observed increase in *Stenotrophomonas* abundance under HS may not be incidental but could play a functional role in mediating inflammatory and endocrine responses, thereby contributing to elevated levels of IL-6 and cortisol. This complex interplay suggests a multifactorial pathophysiological response: HS initiates an endocrine reaction leading to increased cortisol, which, while initially adaptive, can result in immune suppression with prolonged exposure. Simultaneously, elevated IL-6 levels indicate sustained inflammatory signaling. This combination may disrupt microbial homeostasis in the rumen, promoting the growth of opportunistic pathogens like *Stenotrophomonas*. In support of this, previous studies have demonstrated that HS can alter gut microbial composition, resulting in downstream physiological effects [11]. Notably, the present study also found a correlation between *Pseudomonas* abundance and body temperature, suggesting a thermosensitive microbial response. Additionally, associations between specific microbes, such as *Aerococcus*,* Arthrobacter*, and *Carnobacterium*, and host metabolic markers (e.g., AST activity and plasma triglyceride levels) were observed. Although the functional implications of these associations remain unclear, these findings suggest potential microbial biomarkers or modulators of metabolic responses during HS. *Aerococcus*, although rarely described in the rumen, is recognized as an opportunistic pathogen and has been observed in higher abundance in cows supplemented with herbal feed additives [131]. *Arthrobacter* has been linked to animals with low residual feed intake, indicating possible metabolic efficiency [132]. *Carnobacterium* has previously been associated with HS in beef cattle [23] and Holstein cows [133], aligning with findings of the current study. Among protozoa, *Entodinium* exhibited positive correlations with HSP-90, AST, and TNF-α. However, evidence supporting direct biological interactions between *Entodinium* and host biomarkers is limited. Therefore, this association is more likely to reflect a shared response to changes in rumen physiology driven by temperature-related stress rather than a direct host–protozoan interaction. Taken together, these data highlight a significant association between rumen microbial dynamics and physiological stress responses, suggesting that environmental HS not only elicits host responses but also reshapes the microbial ecology of the rumen. This microbial shift may, in turn, exacerbate systemic responses to HS, forming a feedback loop that further impacts animal health and productivity.

## Conclusions

This study investigated the complex interplay between the host and rumen microbiome in zebu calves subjected to HS, aiming to understand how these interactions contribute to thermal resilience. The study revealed that HS significantly impacted calves physiologically, leading to increased body temperature, respiratory rate, and elevated stress markers such as cortisol and IL-6. Despite these physiological challenges, the study observed remarkable stability in volatile fatty acid concentrations, suggesting that the rumen’s crucial fermentative function remained largely intact. At the microbial level, HS induced notable shifts in rumen bacterial, fungal, archaeal, and protozoal communities. For instance, there was an increase in specific bacterial groups, such as Pseudomonadota, and fungal genera, such as *Thermothielavioides*, under HS, whereas other groups, such as bacterial phylum Bacillota and fungi *Schizosaccharomyces*, were more prevalent under TN condition. In addition, several Methanobrevibacter species were enriched in both groups. From a protozoan perspective, the relative abundance of *E. bursa* and *E. longinucleatum* was higher in the TN group than in the HS group. These microbial shifts were not isolated, and the study found strong correlations between specific microbial taxa and host physiological responses. For example, *Stenotrophomonas* was positively linked to stress indicators, whereas *Aerococcus* was linked to other physiological parameters. In essence, these findings underscore the critical role of the rumen microbiome in helping zebu calves cope with HS. Despite the physiological strain and alterations in microbial composition, the maintenance of key metabolic functions, such as VFA production, and the intricate connections between the host stress responses and the microbial community highlight the adaptive capacity of the microbiome and its contribution to the ability of calves to withstand challenging thermal conditions.

## Supplementary Information

Below is the link to the electronic supplementary material.

Supplementary Material 1

## Data Availability

The Raw sequences were submitted to the NCBI SRA database under Bioperoject no. PRJNA1096733. The genome sequences of all MAG were submitted to NCBI genome database (SRR29372413- SRR29372423). All MAG sequences are available at NCBI genome database ( https://www.ncbi.nlm.nih.gov/datasets/genome/?bioproject=PRJNA1096733 ).
