# Supplementary material for: Host–microbiome interplay supports heat stress resilience in zebu calves
Source: Anim Microbiome. 2026 Mar 3;8:39. doi: 10.1186/s42523-026-00539-8 (PMC13064310; doi:10.1186/s42523-026-00539-8)
Supplement: Supplementary file 1 — Supplementary Material 1 [file 42523_2026_539_MOESM1_ESM.docx]

**Host–microbiome interplay supports heat stress resilience in zebu calves**

Brijesh Yadav^1,*^, Goutam Banerjee^2^, Anandita Srivastava^1^, Arun Kumar Madan^1^, Ravindra Kumar^3^, Pratik Banerjee^2,*^

^1^ Prof. M.D. Pandey Bio-Climatology Laboratory, Department of Veterinary Physiology, College of Veterinary Science and Animal Husbandry, Veterinary University (DUVASU), Mathura, UP, India^; 2^ Food Safety and Molecular Microbiology Laboratory, Department of Food Science and Human Nutrition, University of Illinois Urbana-Champaign, Urbana, IL, USA; ^3^ ICAR-Central Institute for Research on Goats, Farah, Mathura, UP, India

**
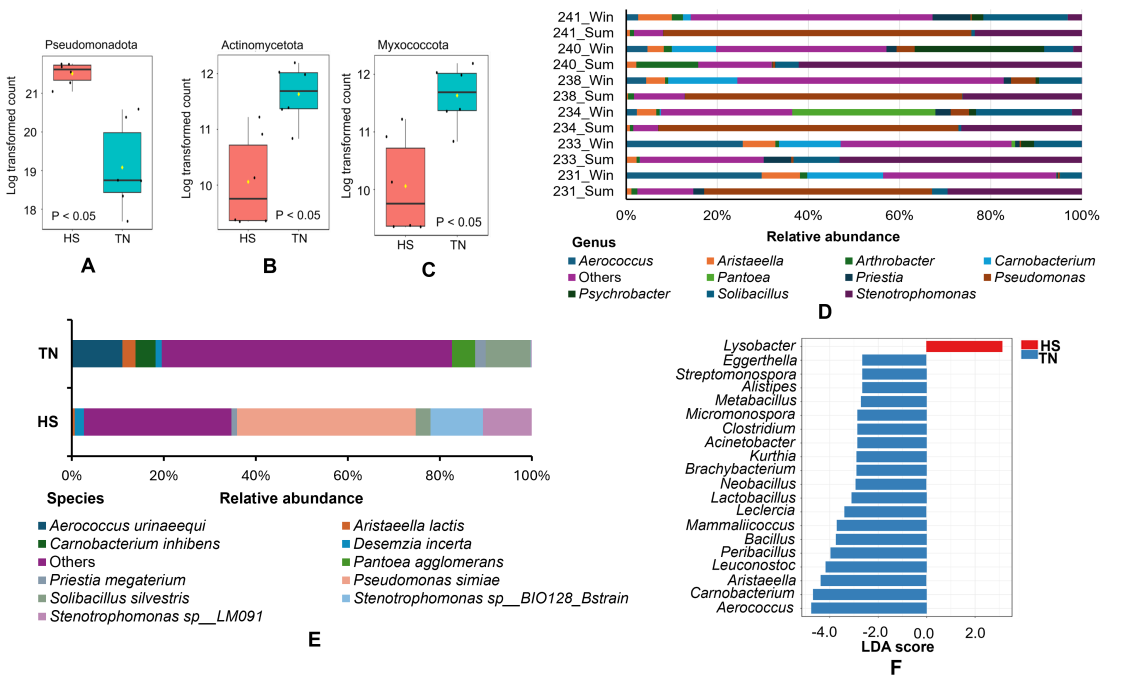
**

**Figure S1.** (A–C) Significant (p < 0.05) differences in the abundance of three bacterial phyla between TN and HS conditions. (D) Relative abundance of bacterial genera across different samples. (E) Relative abundance of species in the TN and HS groups. (F) LEfSe analysis showing significantly enriched bacterial genera (p < 0.05) between the two groups, represented by LDA scores.

**
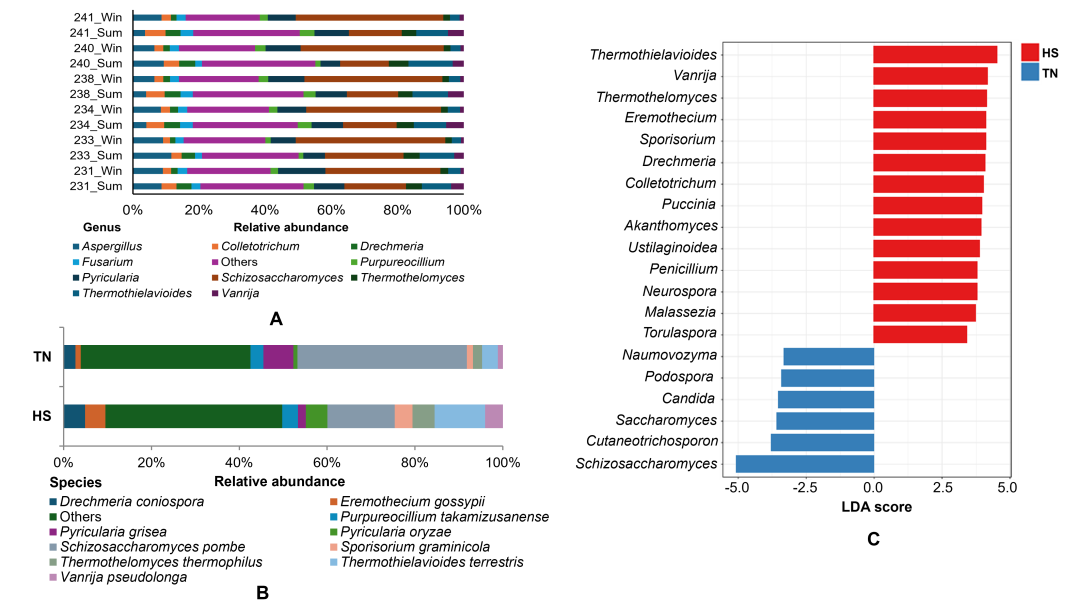
**

**Figure S2.** (A) Relative abundance of fungal genera across different samples. (B) Relative abundance of species in the TN and HS groups. (C) LEfSe analysis showing significantly enriched fungal genera (p < 0.05) between the two groups, represented by LDA scores.


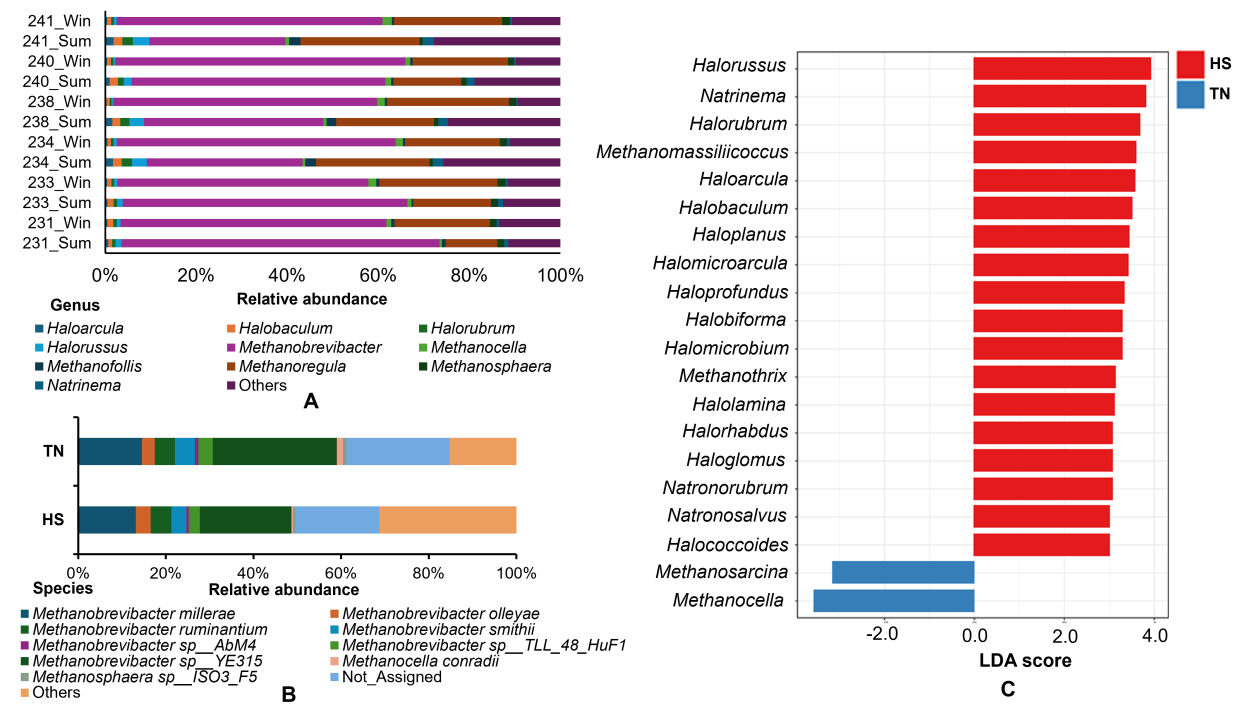


**Figure S3.** (A) Relative abundance of archaeal genera across different samples (B) Relative abundance of species in the TN and HS groups. (C) LEfSe analysis showing significantly enriched archaeal genera (p < 0.05) between the two groups, represented by LDA scores.


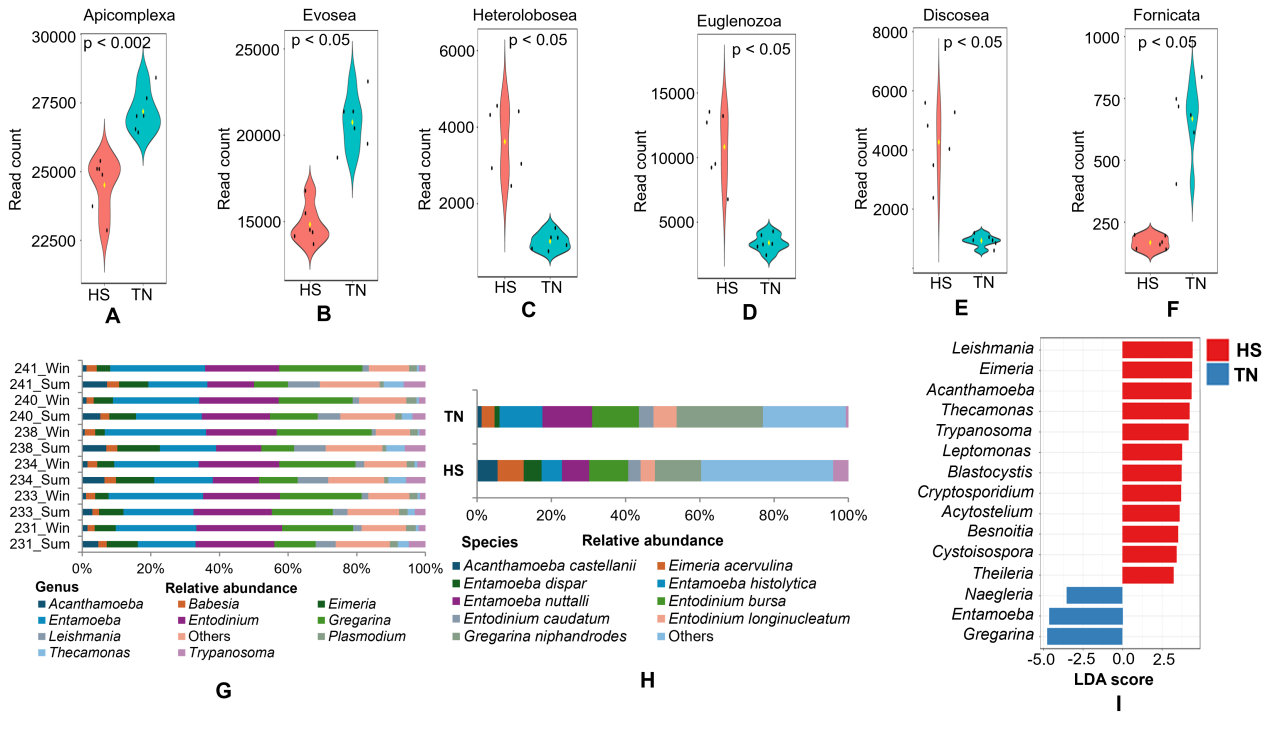


**Figure S4.** (A-F) Significant (p < 0.05) differences in the abundance of protozoan (protists) phyla between TN and HS conditions. Significant differences (Mann–Whitney test) were observed in the abundance of six phyla: Apicomplexa (*p* = 0.002), Evosea (*p* = 0.002), Heterolobosea (*p* = 0.002), Euglenozoa (*p* = 0.002), Discosea (*p* = 0.002), and Fornicata (*p* = 0.004). (G) Relative abundance of protozoan genera across different samples. (H) Relative abundance of species in the TN and HS groups. (I) LEfSe analysis showing significantly enriched protozoan genera (p < 0.05) between the two groups, represented by LDA scores.

**Table S1**. The quality assessment of bacterial MAGs assessed by CheckM.

| **Samples** | **MAGs** | **Bins Statistics** | |
| --- | --- | --- | --- |
|  |  | **Completeness** | **Contamination** |
| 231_Sum | *Pseudomonas simiae*  *Priestia megaterium*  *Solibacillus sronensis*  *Stenotrophomonas* sp.003484865  *Rhodococcus erythropolis* | 99.79  77.88  80.49  97.58  98.95 | 0.099  1.8  0  1.534  0.588 |
| 231_Win | *Aerococcus urinaeequi*  *Psychrobacillus* sp.012843435  *Desemzia incerta*  GCA-900199385 sp.902764875  *Limivicinus* sp.  *Carnobacterium inhibens*  *Leuconostoc mesenteroides*  *Carnobacterium* sp. | 92.79  58.62  80.82  58.27  51.02  73.95  74.88  78.44 | 1.134  1.724  0.546  3.448  2.034  0.193  1.19  3.448 |
| 233_Sum | *Stenotrophomonas* sp. 003484865  *Psychrobacillus psychrodurans*  *Solibacillus* sp.  *Psychrobacillus* sp.  *Desemzia incerta*  *Rhodococcus erythropolis*  *Priestia megaterium*  *Ruminococcus* sp.900317315 | 100  69.31  97.24  88.26  98.9  56.04  89.23  91.93 | 1.534  2.317  0  0.22  2.459  0.742  1.797  1.403 |
| 233_Win | *Aerococcus urinaeequi*  *Psychrobacter alimentarius*  *Carnobacterium* sp.  *Solibacillus* sp.  *Succiniclasticum* sp. | 96.79  87.53  99.45  99  51.96 | 0.824  2.044  3.187  2.373  3.56 |
| 234_Sum | *Stenotrophomonas* sp.003484865  *Arthrobacter* sp.  *Solibacillus* sp.  *Pseudomonas simiae* | 98.81  54.27  100  99.93 | 0.775  1.337  0.883  0.099 |
| 234_Win | *Pseudomonas monteilii*  *Solibacillus isronensis*  *Psychrobacillus* sp.012843435  *Stenotrophomonas maltophilia*  *Aerococcus urinaeequi*  *Priestia megaterium*  GCA-900199385 sp.902764875  *Enterococcus casseliflavus*  *Pantoea agglomerans* | 98.06  84.08  59.82  66.23  94.5  52.7  62.55  60.47  100 | 0.753  1.512  1.724  1.402  1.831  2.481  3.448  2.539  0.191 |
| 238_Sum | *Exiguobacterium indicum*  *Desemzia incerta*  *Stenotrophomonas* sp.003484865  *Pseudomonas simiae*  *Rhodococcus erythropolis* | 96.87  98.9  99.31  99.93  99.74 | 0  1.912  0.844  0.099  0.566 |
| 238_Win | UBA7405 sp.000755535  UBA2862 sp.900318045  *Leuconostoc lactis*  *Carnobacterium inhibens*  *Psychrobacillus psychrotolerans*  *Desemzia incerta*  *Pseudomonas atacamensis*  *Leuconostoc mesenteroides*  *Aerococcus urinaeequi*  *Solibacillus* sp.  *Limivicinus* sp. | 74.06  56.36  99.4  98.9  98.83  83.92  94.91  100  82  99.33  65.22 | 2.495  2.446  0  0.865  0.662  0.728  0.225  0  0.549  1.545  2.498 |
| 240_Sum | *Arthrobacter sp.*  *Rhodococcus erythropolis*  *Solibacillus sp.*  CAG-791 sp.902794825  *Ruminococcus* sp.900320415  *Stenotrophomonas* sp.003484865  *Sporosarcina* sp.013408665  UBA1066 sp.900314565  *Saccharopolyspora rectivirgula*  *Niallia* sp.  *Alkalibacterium* sp. | 90.39  81.03  100  67.76  92.21  99.22  97.35  72.9  60  79.36  71.69 | 1.441  1.296  0  1.301  1.006  1.189  0.662  1.729  2.364  1.709  3.187 |
| 240_Win | *Psychrobacter alimentarius*  *Solibacillus* sp.  *Methanobrevibacter* sp.  *Mammaliicoccus vitulinus*  GCA-900199385 sp.900320755  *Aerococcus urinaeequi*  *Exiguobacterium indicum*  *Pseudomonas qingdaonensis*  *Psychrobacillus* sp.012843435  *Carnobacterium inhibens*  *Solibacillus silvestris* | 91.74  97.05  77.39  99.35  57.17  94.23  98.02  96.75  81.41  62.06  81.76 | 0.961  0.883  0  1.104  4.385  0.274  0  0.516  4.635  0  1.324 |
| 241_Sum | *Lysinibacillus* sp.  *Arthrobacter* sp.  *Solibacillus* sp.  *Enterococcus casseliflavus*  *Stenotrophomonas* sp.003484865  *Pseudomonas simiae*  *Rhodococcus erythropolis* | 78.54  86.84  99.44  97.04  99.13  99.93  99.93 | 2.373  2.435  0  1.32  0.844  0.099  0.521 |
| 241_Win | *Solibacillus* sp.  *Solibacillus* sp.  *Priestia megaterium*  *Psychrobacter alimentarius*  *Desemzia incerta*  *Aerococcus urinaeequi*  *Solibacillus isronensis*  *Enterococcus casseliflavus* | 98.67  96.07  57.11  74.45  90.71  82.77  68.42  95.34 | 2.207  1.545  0.228  1.52  2.459  2.618  1.754  0.754 |

**Table S2.** The relative abundance of bacterial MAGs in respective samples.

| **Sample** | **MAGs** | **Abundance (%)** |
| --- | --- | --- |
| 231_Sum | *Pseudomonas simiae*  *Priestia megaterium*  *Solibacillus sronensis*  *Stenotrophomonas* sp.003484865  *Rhodococcus erythropolis* | 26.073263  1.541736  4.095972  26.066662  2.1403105 |
| 231_Win | *Aerococcus urinaeequi*  *Psychrobacillus* sp.012843435  *Desemzia incerta*  GCA-900199385 sp.902764875  *Limivicinus* sp.  *Carnobacterium inhibens*  *Leuconostoc mesenteroides*  *Carnobacterium* sp. | 7.7727265  0.4207959  0.52957475  0.97573817  0.7617507  2.392464  0.3955916  1.7533551 |
| 233_Sum | *Stenotrophomonas* sp. 003484865  *Psychrobacillus psychrodurans*  *Solibacillus* sp.  *Psychrobacillus* sp.  *Desemzia incerta*  *Rhodococcus erythropolis*  *Priestia megaterium*  *Ruminococcus* sp.900317315 | 27.019567  0.9343406  6.819354  1.4019445  9.576672  0.65572023  1.9407841  1.2835032 |
| 233_Win | *Aerococcus urinaeequi*  *Psychrobacter alimentarius*  *Carnobacterium* sp.  *Solibacillus* sp.  *Succiniclasticum* sp. | 8.911712  0.65805656  4.165593  7.9662805  1.3158042 |
| 234_Sum | *Stenotrophomonas* sp.003484865  *Arthrobacter* sp.  *Solibacillus* sp.  *Pseudomonas simiae* | 29.696072  1.0887164  3.3594668  42.91518 |
| 234_Win | *Pseudomonas monteilii*  *Solibacillus isronensis*  *Psychrobacillus* sp.012843435  *Stenotrophomonas maltophilia*  *Aerococcus urinaeequi*  *Priestia megaterium*  GCA-900199385 sp.902764875  *Enterococcus casseliflavus*  *Pantoea agglomerans* | 1.0692987  8.698883  0.96377367  0.834618  1.7681278  0.9351542  1.7544388  0.68020386  8.656026 |
| 238_Sum | *Exiguobacterium indicum*  *Desemzia incerta*  *Stenotrophomonas* sp.003484865  *Pseudomonas simiae*  *Rhodococcus erythropolis* | 4.3044643  2.9800396  29.95699  40.756725  1.4788294 |
| 238_Win | UBA7405 sp.000755535  UBA2862 sp.900318045  *Leuconostoc lactis*  *Carnobacterium inhibens*  *Psychrobacillus psychrotolerans*  *Desemzia incerta*  *Pseudomonas atacamensis*  *Leuconostoc mesenteroides*  *Aerococcus urinaeequi*  *Solibacillus* sp.  *Limivicinus* sp. | 0.52139163  1.159513  2.9948907  5.998737  1.8502764  1.1512069  0.87055266  4.5279217  2.0206401  16.358473  1.3298994 |
| 240_Sum | *Arthrobacter sp.*  *Rhodococcus erythropolis*  *Solibacillus sp.*  CAG-791 sp.902794825  *Ruminococcus* sp.900320415  *Stenotrophomonas* sp.003484865  *Sporosarcina* sp.013408665  UBA1066 sp.900314565  *Saccharopolyspora rectivirgula*  *Niallia* sp.  *Alkalibacterium* sp. | 9.079528  0.36732137  3.674117  0.4940443  0.6217255  32.645176  0.522799  0.398049  0.3442658  0.57399446  0.3158493 |
| 240_Win | *Psychrobacter alimentarius*  *Solibacillus* sp.  *Methanobrevibacter* sp.  *Mammaliicoccus vitulinus*  GCA-900199385 sp.900320755  *Aerococcus urinaeequi*  *Exiguobacterium indicum*  *Pseudomonas qingdaonensis*  *Psychrobacillus* sp.012843435  *Carnobacterium inhibens*  *Solibacillus silvestris* | 10.936842  3.5231295  1.1438336  1.4235772  1.3734382  2.8231533  4.036168  0.8421997  0.9875208  2.6917596  1.8038901 |
| 241_Sum | *Lysinibacillus* sp.  *Arthrobacter* sp.  *Solibacillus* sp.  *Enterococcus casseliflavus*  *Stenotrophomonas* sp.003484865  *Pseudomonas simiae*  *Rhodococcus erythropolis* | 0.41015562  1.3693303  1.1004678  0.6073364  27.502138  46.42973  1.2435013 |
| 241_Win | *Solibacillus* sp.  *Solibacillus* sp.  *Priestia megaterium*  *Psychrobacter alimentarius*  *Desemzia incerta*  *Aerococcus urinaeequi*  *Solibacillus isronensis*  *Enterococcus casseliflavus* | 8.987754  5.096387  1.2613897  0.7234886  1.2689661  1.0978554  3.6632092  1.5494281 |

**Table S3.** The annotation and detail information of bacterial MAGs assessed by Prokka.

| **Samples** | **MAGs** | **Annotation** | | | | | |
| --- | --- | --- | --- | --- | --- | --- | --- |
|  |  | **CDS** | **Gene** | **Misc RNA** | **rRNA** | **tRNA** | **tmRNA** |
| 231_Sum | *Pseudomonas simiae*  *Priestia megaterium*  *Solibacillus sronensis*  *Stenotrophomonas* sp.003484865  *Rhodococcus erythropolis* | 5594  2706  2751  3827  6379 | 5722  2820  2851  3906  6467 | 69  72  71  19  35 | 1  5  2  1  1 | 57  35  27  58  51 | 1  2  0  1  1 |
| 231_Win | *Aerococcus urinaeequi*  *Psychrobacillus* sp.012843435  *Desemzia incerta*  GCA-900199385 sp.902764875  *Limivicinus* sp.  *Carnobacterium inhibens*  *Leuconostoc mesenteroides*  *Carnobacterium* sp. | 1433  1364  1314  1883  1538  1337  1073  2945 | 1555  1419  1350  1909  1563  1376  1118  3011 | 29  37  25  8  12  32  22  47 | 5  0  1  1  0  0  0  1 | 88  18  9  17  13  7  22  17 | 0  0  1  0  0  0  1  1 |
| 233_Sum | *Stenotrophomonas* sp. 003484865  *Psychrobacillus psychrodurans*  *Solibacillus* sp.  *Psychrobacillus* sp.  *Desemzia incerta*  *Rhodococcus erythropolis*  *Priestia megaterium*  *Ruminococcus* sp.900317315 | 3937  3606  3768  3056  2173  4990  2800  2170 | 4014  3686  3881  3162  2295  5031  2879  2234 | 19  72  91  64  46  19  71  20 | 1  2  7  2  4  0  0  6 | 56  5  14  39  71  21  7  37 | 1  1  1  1  1  1  1  1 |
| 233_Win | *Aerococcus urinaeequi*  *Psychrobacter alimentarius*  *Carnobacterium* sp.  *Solibacillus* sp.  *Succiniclasticum* sp. | 1441  2111  2539  3742  1232 | 1509  2145  2630  3856  1302 | 27  6  47  85  18 | 1  0  1  9  2 | 40  27  42  19  49 | 0  1  1  1  1 |
| 234_Sum | *Stenotrophomonas* sp.003484865  *Arthrobacter* sp.  *Solibacillus* sp.  *Pseudomonas simiae* | 3728  1994  3869  5601 | 3812  2026  3996  5725 | 19  12  90  70 | 0  0  4  1 | 64  18  32  52 | 1  2  1  1 |
| 234_Win | *Pseudomonas monteilii*  *Solibacillus isronensis*  *Psychrobacillus* sp.012843435  *Stenotrophomonas maltophilia*  *Aerococcus urinaeequi*  *Priestia megaterium*  GCA-900199385 sp.902764875  *Enterococcus casseliflavus*  *Pantoea agglomerans* | 4283  2008  1973  2600  1638  1630  3158  1401  4422 | 4387  2076  2033  2636  1695  1712  3188  1425  4573 | 59  49  46  12  31  58  10  22  99 | 1  2  0  2  5  3  0  0  1 | 43  17  13  12  20  19  19  2  50 | 1  0  1  0  1  2  1  0  1 |
| 238_Sum | *Exiguobacterium indicum*  *Desemzia incerta*  *Stenotrophomonas* sp.003484865  *Pseudomonas simiae*  *Rhodococcus erythropolis* | 2926  2151  3755  2830  6377 | 3010  2261  3833  2941  6462 | 52  49  19  16  33 | 9  4  0  2  0 | 23  56  58  33  51 | 0  1  1  1  1 |
| 238_Win | UBA7405 sp.000755535  UBA2862 sp.900318045  *Leuconostoc lactis*  *Carnobacterium inhibens*  *Psychrobacillus psychrotolerans*  *Desemzia incerta*  *Pseudomonas atacamensis*  *Leuconostoc mesenteroides*  *Aerococcus urinaeequi*  *Solibacillus* sp.  *Limivicinus* sp. | 3389  1734  1495  2229  3638  1643  4897  1754  1381  4016  2264 | 3513  1758  1547  2329  3755  1689  5017  1836  1429  4148  2308 | 78  12  20  48  94  34  77  35  22  83  15 | 1  0  1  10  2  1  1  1  1  4  1 | 44  11  30  41  20  10  41  45  25  44  28 | 1  1  1  1  1  1  1  1  0  1  0 |
| 240_Sum | *Arthrobacter sp.*  *Rhodococcus erythropolis*  *Solibacillus sp.*  CAG-791 sp.902794825  *Ruminococcus* sp.900320415  *Stenotrophomonas* sp.003484865  *Sporosarcina* sp.013408665  UBA1066 sp.900314565  *Saccharopolyspora rectivirgula*  *Niallia* sp.  *Alkalibacterium* sp. | 3187  6412  3736  1672  1739  3759  2852  1475  2495  3830  1295 | 3254  6473  3857  1730  1778  3839  2948  1510  2535  3936  1333 | 19  29  89  10  12  19  66  7  16  85  29 | 1  0  28  0  3  1  2  0  1  4  0 | 45  32  1  48  23  59  27  26  23  17  9 | 2  0  0  0  1  1  1  2  0  0  0 |
| 240_Win | *Psychrobacter alimentarius*  *Solibacillus* sp.  *Methanobrevibacter* sp.  *Mammaliicoccus vitulinus*  GCA-900199385 sp.900320755  *Aerococcus urinaeequi*  *Exiguobacterium indicum*  *Pseudomonas qingdaonensis*  *Psychrobacillus* sp.012843435  *Carnobacterium inhibens*  *Solibacillus silvestris* | 2180  3559  1519  2199  2243  1455  2695  4813  4839  1715  3117 | 2227  3674  1544  2253  2265  1510  2764  4930  1974  1752  3219 | 9  76  2  34  12  34  51  64  71  30  84 | 0  6  0  1  0  0  0  1  8  1  8 | 37  32  23  18  10  21  17  51  55  6  10 | 1  1  0  1  0  0  1  1  1  0  0 |
| 241_Sum | *Lysinibacillus* sp.  *Arthrobacter* sp.  *Solibacillus* sp.  *Enterococcus casseliflavus*  *Stenotrophomonas* sp.003484865  *Pseudomonas simiae*  *Rhodococcus erythropolis* | 2423  3198  3628  2915  3749  5595  6468 | 2475  3265  3742  2988  3826  5720  5660 | 42  16  89  48  19  70  35 | 1  2  2  1  1  1  1 | 9  47  22  23  56  53  55 | 0  2  1  1  1  1  1 |
| 241_Win | *Solibacillus* sp.  *Solibacillus* sp.  *Priestia megaterium*  *Psychrobacter alimentarius*  *Desemzia incerta*  *Aerococcus urinaeequi*  *Solibacillus isronensis*  *Enterococcus casseliflavus* | 3837  3767  1161  1635  1807  1327  2474  2688 | 3965  3872  1202  1658  1886  1368  2553  2749 | 87  83  35  6  42  25  61  43 | 6  1  1  0  3  0  2  0 | 34  20  5  16  33  16  14  17 | 1  1  0  1  1  0  2  1 |
